# Performance of the LIAISON PLEX gram-negative blood culture assay for identifying bacterial pathogens and resistance genes in blood cultures

**DOI:** 10.1128/jcm.00018-26

**Published:** 2026-05-22

**Authors:** Neelam Dhiman, Christopher L. Emery, Siu-Kei Chow, Dooil Ho, Paul Granato, Brian Bernier, Janet Farhang

**Affiliations:** 1 Quest Diagnostics, Lewisville, Texas, USA; 2 Indiana University Health 22529, Indianapolis, Indiana, USA; 3 MultiCare Health System 14458, Tacoma, Washington, USA; 4 Laboratory Alliance of Central New York 51972 , Liverpool, New York, USA; 5 Luminex Corporation 17737 , Austin, Texas, USA; Children's Hospital Los Angeles, Los Angeles, California, USA

**Keywords:** gram-negative bacteria, antimicrobial resistance marker, blood culture, *in vitro *diagnostic assay, sample-to-answer

## Abstract

Gram-negative bacteria cause potentially life-threatening bloodstream infections worldwide. Delayed initiation of optimal antimicrobial therapy worsens health outcomes. Molecular assays characterize bloodstream pathogens and their antimicrobial resistance (AMR) genes faster than gold-standard culture-based methods. This multi-site study evaluated the performance of the automated multiplex LIAISON PLEX Gram-Negative Blood Culture (BCN) Assay (Luminex Corporation), which tests positive blood cultures for 19 gram-negative bacteria and 8 AMR genes, in approximately 2 h. Samples included 351 prospectively collected, 231 pre-selected, and 746 contrived specimens. For combined prospective plus pre-selected specimens, sensitivity/positive percent agreement was 90.0%–100% for 18/19 on-panel bacterial targets, and 97.8%–100% for detected AMRs. Specificity/negative percent agreement was ≥99.3% for all 19 bacterial targets, and ≥99.6% for AMRs. Common pathogens in prospective samples were *Escherichia coli* (51.4%), *Klebsiella pneumoniae* (12.9%), and *Pseudomonas* spp. (8.3%). Contrived specimen sensitivity and specificity both averaged >99.8% across all assay targets. All 13 tested bottle/matrix types gave 100% detection of 21 targetable pathogens tested. The assay identified a cumulative 246 reportable on-target strains, indicating broad inclusivity. Cross-reactivity was observed with 17/41 off-panel gram-negative bacteria; no cross-reactivity was observed with 61 gram-positive bacteria or 11 fungal pathogens. Low concentrations of 10 on-panel bacteria were detectable alongside high concentrations of 9 other on-panel targets or 7 gram-positive potential confounders. Six potential interfering substances in blood culture bottles had no impact on the detection of four representative on-target bacteria. Reproducibility approached 100% both within-lab and across sites. The LIAISON PLEX BCN Assay provides excellent sensitivity and specificity for quickly characterizing gram-negative bacterial pathogens and their resistance genes in positive blood culture specimens.

**IMPORTANCE:**

Bloodstream bacterial infections may cause serious illness and death. Successful treatment demands rapid pathogen identification so the optimal antibiotic therapy can be initiated. Blood culture is standard for confirming bloodstream infection but may not provide detailed pathogen information in a timely manner. The LIAISON PLEX Gram-Negative Blood Culture assay quickly analyzes blood cultures to identify 19 pathogenic gram-negative bacteria and 8 associated antibiotic drug resistance genes. The LIAISON PLEX BCN Assay demonstrated a high degree of reliability in rapidly characterizing the infective pathogen and associated resistance genes in blood cultures from 582 patients with demonstrated gram-negative bloodstream infection. Assay performance was confirmed in 746 contrived samples in which samples were spiked with different gram-negative bacteria.

## INTRODUCTION

Bloodstream infection (BSI) with bacteria carries a substantial risk of morbidity and mortality, and the ongoing emergence of antimicrobial resistance (AMR) genes within infective bacteria is concerning. Globally, an estimated 5 million annual deaths are associated with or directly attributable to BSIs by antibiotic-resistant bacteria (1); in the U.S. alone, there are an estimated 1.7 million annual hospitalizations for bacterial sepsis and 350,000 deaths (2). Gram-negative bacteria have increased in importance in BSI over recent decades, rising as the causative agent from 33% to 43% of worldwide bacteremia cases between 1997 and 2016 (3). Clinicians increasingly encounter antibiotic-resistant bacteria in patients, with expectedly worse clinical outcomes. For instance, deaths attributable to carbapenem-resistant gram-negative bacteria have increased by 70% globally between 1990 and 2021 (1). Rapid and accurate identification of the causative pathogen(s) and their AMR genes in BSI is essential for quickly initiating the most appropriate antimicrobial therapy. The survival rate of patients with sepsis drops by almost 8% for each hour that optimal treatment is delayed (4).

Blood culture is the gold-standard method for diagnosing BSI but may require several days to confirm infection, identify the causative agent(s), and predict AMR characteristics (5). Newer molecular assays have been developed that more rapidly characterize BSI than microbial growth-based techniques (6). US Food and Drug Administration-cleared DNA microarray-based (e.g*.,* Verigene, Luminex Corp., Austin, TX; previously Nanosphere) and/or nucleic acid amplification-based (e.g., FilmArray system, bioMérieux, Marcy l’Etoile, France; previously BioFire Diagnostics; and cobas ePlex, Roche Diagnostics USA, Indianapolis, IN) methods simultaneously detect gram-negative bacteria and their cephalosporin- and carbapenem-based AMR genes directly from positive blood cultures (5, 6). However, pathogen and AMR coverage varies by assay, and several clinically relevant targets may be missed. The Verigene Gram-Negative Blood Culture Kit, the first non-amplifying array-based molecular multiplex gram-negative assay to receive FDA clearance and the predecessor to the current study assay, was designed to detect eight pathogenic gram-negative bacterial species/genera and six AMR genes in under 2.5 h (7).

The LIAISON PLEX Gram-Negative Blood Culture (BCN) Assay (Luminex Corporation) is a broad-coverage, DNA array-based assay that interrogates positive blood cultures to qualitatively establish the presence or absence of 19 pathogenic gram-negative bacterial species/families that are responsible for most gram-negative BSIs (1, 8) and 8 clinically relevant AMR genes. By comparison, the FilmArray and cobas ePlex assays include 15 and 21 gram-negative bacterial targets and 6 and 7 gram-negative-associated AMR genes, respectively. FilmArray simultaneously evaluates gram-negative, gram-positive, and yeast targets. The ePlex assay has the broadest Enterobacterales coverage. Both the ePlex and LIAISON PLEX assays may potentially reduce costs by using a Gram staining-directed workflow and selective target testing. All produce results within 1–2 h.

Pathogen targets of the LIAISON PLEX BCN Assay include the three gram-negative bacterial groups designated as “critical priority” in the World Health Organization’s updated 2024 list of dangerous bacterial pathogens (8). These are also AMR infectious agents identified as urgent public health hazards in the U.S. Centers for Disease Control and Prevention’s 2024 Antimicrobial Resistance Threats update (9). The automated multiplex assay requires only 2 min of hands-on time to produce pathogen and AMR results in approximately 2 h, which is much faster than conventional culture-based pathogen and susceptibility testing that often requires several additional days after initial blood culture positivity. The current report details our comprehensive assessment of the clinical and analytical performance characteristics of the LIAISON PLEX BCN Assay.

## MATERIALS AND METHODS

### LIAISON PLEX gram-negative blood culture assay

The FDA-cleared LIAISON PLEX BCN Assay (FDA Clearance #K243013) is a qualitative, multiplexed, direct hybridization (no amplification) nucleic acid-based diagnostic assay that simultaneously interrogates positive blood culture specimens for 26 targets comprising 19 gram-negative bacterial family/genera/species and 8 related AMR genes that are causative for BSIs (10) (Table 1). Sample hybridization uses a cDNA microarray capture format coupled with proprietary gold nanoparticle-based NanoGrid detection chemistry (Luminex). Bacterial targets in the LIAISON PLEX BCN Assay are shown in Table 1.

**TABLE 1:** The LIAISON PLEX Gram-Negative Blood Culture Assay simultaneously interrogates blood cultures showing gram-negative bacterial staining for the presence of 19 bacterial targets and 8 antimicrobial resistance genes

Gram-negative genera and species	Resistance markers
*Enterobacteriaceae/Morganellaceae*	CTX-M (*bla*_CTX-M_)
*Acinetobacter baumannii* ^a^	IMP (*bla*_IMP_)
*Acinetobacter* spp.	KPC (*bla*_KPC_)
*Citrobacter* spp.	NDM (*bla*_NDM_)
*Enterobacter* spp.^b^	OXA (*bla*_OXA_)^d^
*Escherichia coli* ^c^	VIM (*bla*_VIM_)
*Haemophilus influenzae*	MCR^e^
*Klebsiella oxytoca*	SME (*bla*_SME_)
*Klebsiella pneumoniae*	
*Klebsiella variicola*	
*Morganella morganii*	
*Neisseria meningitidis*	
*Proteus* spp.	
*Pseudomonas aeruginosa*	
*Pseudomonas* spp.	
*Salmonella* spp.	
*Serratia marcescens*	
*Stenotrophomonas maltophilia*	

^a^ LIAISON PLEX BCN Assay’s *A. baumannii* species-specific hybridization sequences capture only *A. baumannii* and not other *A. baumannii* complex members.

^b^ Due to reclassification, *Klebsiella aerogenes* will be reported *Enterobacter* spp.

^c^ LIAISON PLEX BCN Assay will not distinguish between *Escherichia coli* and *Shigella* spp. (*S. dysenteriae, S. boydii, S. flexneri,* and *S. sonnei*).

^d^ OXA-23 family, OXA-24/40/143 family, OXA-48 family, and OXA-58 family.

^e^ *mcr-1*, *mcr-2*, *mcr-3*.

The LIAISON PLEX BCN Assay rapidly supplements findings from positive continuous monitoring blood cultures that demonstrate gram-negative-staining bacteria. The LIAISON PLEX BCN assay is intended to augment other clinical and laboratory findings for diagnosing bacterial bloodstream infections. Sample-to-result time is approximately 2 h using the automated LIAISON PLEX System (Luminex) that performs multiple assay steps, including nucleic acid extraction, hybridization, and signal analysis within a single disposable cartridge (11). For each LIAISON PLEX instrument, up to six assay cartridges can be processed concurrently in a random-access format, that is, cartridges can be added mid-run without interrupting an ongoing analysis. Direct hybridization with non-amplified bacterial DNA reduces the likelihood of false-positive calls that may occur by amplifying DNA from dead or non-viable bacteria that are common in blood cultures (12) and also eliminates the additional reagent costs of amplification.

Concisely, broth samples from blood cultures containing gram-negative-staining bacteria are transferred into a single-use assay cartridge, the Sample and Cartridge ID barcodes are scanned, and the cartridge is loaded into the LIAISON PLEX System, where the analysis is initiated according to the pre-specified protocol. Sample processing entails the following: (i) nucleic acid extraction using paramagnetic beads, (ii) incubation of isolated nucleic acids with target-specific DNA sequences in microarray format, (iii) detection of captured target-specific sequences by array hybridization with targeted gold-conjugated oligomer probes, (iv) signal amplification with colloidal silver, and (iv) assessment of the signal at array spots to qualitatively determine the presence or absence of each target. The resistance gene is reported as “Not Reviewed” if the associated organism(s) are not concurrently detected. Logical flow for interpreting LIAISON PLEX BCN organism and associated AMR results is outlined in Table S1.

Internal controls present within the LIAISON PLEX BCN Assay cartridge ensure sample preparation and detection performance. Verified negative blood matrix (NBM) can be used as a negative control, and previously characterized positive samples, commercially available controls, or verified NBM spiked with well-characterized organisms can serve as external positive controls.

### Clinical performance

Diagnostic accuracy of the LIAISON PLEX BCN Assay was evaluated using 381 specimens prospectively collected between March and July 2024, from four geographically diverse clinical sites across the USA that met the pre-determined study inclusion criteria. Samples were remnant, de-identified, blood culture specimens from patients exhibiting clinical signs and symptoms of bloodstream infections, with positive identification using a continuous monitoring blood culture system and exhibiting gram-negative morphology on Gram stain. Clinical runs and re-runs using the LIAISON PLEX BCN Assay were tested on the LIAISON PLEX System by trained operators at four clinical sites. Prospectively collected specimens were allowed to be stored for ≤3 days at 2°C–8°C or ≤30 days frozen at −70°C after initial blood culture result and prior to LIAISON PLEX BCN testing (10). The prospective samples contained 18 assay targets.

Reference testing methods included the VITEK 2 system (bioMérieux, Marcy-l’Etoile, FR) (13) and PCR/bidirectional sequencing (BDS). The primary comparator was VITEK 2 for most organisms, but because VITEK 2 does not perform well for some organisms, BDS was used as the reference method for *Klebsiella variicola*, *Acinetobacter baumannii*, *Klebsiella oxytoca, Klebsiella pneumoniae*, and the AMR genes, and was also used for resolving discrepancies between the LIAISON PLEX BCN Assay and VITEK 2. For secondary exploratory analyses, the available standard-of-care data from the predecessor Verigene Gram-Negative Blood Culture Test and/or MALDI-TOF MS that were provided by the clinical sites were also included for comparison. MALDI-TOF MS was performed on subcultured specimens following culture bottle positivity, as per the manufacturer’s instructions.

Continuous monitoring of blood culture systems included the BacT/ALERT VIRTUO, BacT/ALERT 3D (bioMérieux SA; Marcy-l'Étoile, France), and BD BACTEC FX (Becton, Dickinson and Company, Franklin Lakes, NJ).

For targets with low prevalence rates in the prospective study, the prospective specimen set was supplemented with 231 pre-selected, left-over, de-identified specimens obtained from seven commercial vendors in the USA and one site in Italy. Pre-selected specimens contained a cumulative 21 LIAISON PLEX BCN Assay targets identified by standard-of-care testing and confirmed as positive by VITEK 2 and/or PCR, followed by BDS, according to the reference method algorithm prior to study enrollment. Pre-selected samples were stored frozen at −70°C (for 3 months to 4 years 10 months) prior to study enrollment and testing. To minimize bias, pre-selected specimens were tested in a randomized, blinded manner along with negative specimens across testing sites.

Contrived specimens were tested to supplement the positive clinical specimens in the prospective and pre-selected study cohorts. In total, 746 specimens cumulatively representing 25 LIAISON PLEX BCN Assay targets were contrived, blinded, randomized, and assayed along with negative specimens at four testing sites during April to August, 2024.

This study was conducted under the oversight of the Institutional Review Boards of all participating sites and conformed to the tenets of the Declaration of Helsinki and the Health Insurance Portability and Accountability Act (HIPAA) of 1996.

### Bidirectional sequence analysis

BDS analysis was used as the reference method for the AMR genes and for *Klebsiella variicola*, *A. baumannii*, *K. oxytoca*, and *K. pneumoniae* and was also used for discordant resolution. Two analytically validated fragment analysis PCR assays were performed, followed by BDS assays as a composite comparator. Primers used for PCR/BDS targeted the same genomic regions as those targeted by the LIAISON PLEX BCN Assay for each target; however, the primers used for BDS either flanked the assay target or overlapped with the assay target and were different than the primer binding regions in the LIAISON PLEX BCN Assay. Samples were sequenced on an ABI DNA Analyzer 3730xl system using the Sanger (i.e., dideoxy or chain termination) method.

### Analytical performance overview

The LIAISON PLEX BCN Assay was assessed for compatibility with diverse commercial culture bottles/matrices, detectability of multiple target variants, potential interfering substances, possible impact of bacterial co-infections as interferents, cross-reactivity with off-panel organisms, and site-to-site reproducibility.

Reference bacterial cultures were from the American Type Culture Collection (ATCC; Manassas, VA)**,** International Health Management Associates repository (IHMA; Schaumburg, IL), and the UK National Collection of Type Cultures (NCTC; Salisbury, UK).

### Target detection

A Growth and Detection study evaluated the LIAISON PLEX BCN Assay detection of species/targets in blood cultures. The automated blood culture system monitors bottles at ~10 min intervals and signals positive microbial growth via an audible alarm and display message. We refer to this alert as “ring-positive.” Organism concentrations were evaluated at initial ring-positivity and ring-positive +8 h incubation times. Bacterial concentrations were determined as colony-forming units per milliliter (CFU/mL) by culturing aliquots of a bottle-matrix dilution series (diluted in tryptic soy broth) on appropriate agar, and counting resulting colonies.

### Media equivalency

The LIAISON PLEX BCN Assay was evaluated across 12 different types of commercially available blood culture media bottles (in addition to bioMérieux BacT/ALERT FA Plus established as the standard media for the clinical evaluation and for all other analytical studies, e.g., growth and detection and analytical reactivity/inclusivity verification testing), using representative on-panel assay targets with resistance markers (20–63 bottles/matrix type), off-panel gram-positive bacteria (10 bottles/matrix type), and NBM (2 bottles/matrix type).

### Analytical reactivity (inclusivity)

An analytical reactivity study evaluated the inclusivity of the LIAISON PLEX BCN Assay by testing 246 bacterial strains representing on-panel reportable bacteria in triplicate assay cartridges. Up to 39 strains per assay target were included, and strain information is detailed elsewhere (10). Briefly, matrix bottles were inoculated with the tested bacteria and cultured for up to 5 days or until positivity was signaled.

### Analytical specificity (cross-reactivity)

A specificity study evaluated cross-reactivity with the LIAISON PLEX BCN Assay by testing 113 “off-panel” organisms, including both those phylogenetically related to on-panel bacteria and organisms commonly present in blood culture samples. All organisms were grown to blood culture bottle positivity (ring-positive) and tested in triplicate assay cartridges.

### Interfering substances

Performance of the LIAISON PLEX BCN Assay was evaluated in the presence of non-microbial endogenous (unconjugated bilirubin, conjugated bilirubin, hemoglobin, Intralipid lipemia mimetic, and γ-globulin) and exogenous (sodium polyanetholsulfonate, i.e., SPS) potentially interfering substances that may be present in blood culture specimens. Four representative on-panel organisms (*A. baumannii*, *E. coli*, *H. influenzae*, and *N. meningitidis*) were grown to bottle positivity and tested. Each interfering substance was tested across five replicates. A negative control (five replicates) was tested alongside the positive specimens to assess the impact of the same interferents in specimens containing no target. A positive control (specimen without interferents) for all four targets was also tested to verify assay detection fidelity.

### Competitive inhibition/co-infection and microbial interference

To evaluate possible competitive inhibition with the LIAISON PLEX BCN Assay, and performance in identifying co-infections, a set of 10 representative on-panel strains was tested at high concentrations (ring-positive +8 h incubation levels) when combined in a pair-wise fashion with low concentrations (initial ring-positive incubation levels) of each of the other 9 on-panel strains. Each combination was performed in triplicate (*n* = 270 cumulative determinations). The on-panel organisms were chosen to be representative of clinically relevant polymicrobial bloodstream infections. Tested on-panel strains included: *E. cloacae*, *E. coli*, *H. influenzae*, *K. oxytoca*, *K. pneumoniae*, *N. meningitidis*, *P. mirabilis*, *P. aeruginosa*, *A. baumannii*, and *C. freundii*.

To assess the potential off-panel microbial inhibition of the LIAISON PLEX BCN Assay, we evaluated the detectability of low concentrations (ring-positive incubation) of 10 representative on-panel bacteria (same as listed above) in the presence of high concentrations (ring-positive +8 h incubation) of 7 off-panel, clinically relevant, gram-positive bacteria. Determinations were performed in triplicate (*n* = 210 cumulative determinations). The seven potential competitor organisms were chosen because they are commonly found at the sites of bloodstream infections and may be present in positive blood culture samples. Tested gram-positive bacterial strains included *Bacillus cereus*, *Clostridium perfringens*, *Corynebacterium striatum*, *Cutibacterium (Propionibacterium) acnes*, *Staphylococcus aureus*, *Staphylococcus epidermidis*, and *Streptococcus mitis*.

Organism concentrations in CFU/mL for competitive inhibition and microbial interference are shown in Tables S8 and S9, respectively.

### Multi-site and interoperator reproducibility

Site-to-site reproducibility of the LIAISON PLEX BCN Assay was evaluated by performing a reproducibility study using a single lot of BCN Assay cartridge with a designated panel of positive samples by two operators at each of three of the clinical sites. Samples were randomized and blinded to the operators, and runs were performed in triplicate on five non-consecutive days. The reproducibility panel consisted of six representative on-panel bacteria (*A. baumannii*, *K. pneumoniae, H influenzae, N. meningitidis, E. coli,* and *S. marcescens*) representing four AMRs (OXA, KPC, MCR, and SME). The six on-panel organisms were tested at both ring-positive and ring-positive +8 h concentrations. Negative controls included a contrived sample containing an off-panel organism (*Staphylococcus aureus*) cultured to ring-positive +8 h, and an NBM sample. Using the same approach, the reproducibility between the two operators at each site was also compared.

### Statistical analysis

Data are presented as n (%) or value ±95% confidence intervals (CIs), as indicated. Statistical software included SAS v.9.4 (SAS Corp.; Cary, NC) and Excel (Microsoft Corp.). Positive percent agreement (PPA), negative percent agreement (NPA), and accuracy were calculated with 95% CIs using the Wilson method (https://tools.westgard.com/two-by-two-contingency.shtml). Accuracy was defined as the percentage of correct calls (TP + TN) divided by the total calls (TP + TN + FP + FN).

## RESULTS

### Clinical trial participant and specimen details

Demographic information of the patients from whom the 351 prospectively collected and 231 pre-selected specimens included in the study is shown in Table 2.

**TABLE 2:** Demographics and sampling details^*a*^

Parameter	Prospective (*N* = 351) Specimens (%)	Pre-selected (*N* = 231) Specimens (%)
Gender		
Male	181 (51.6%)	136 (58.9%)
Female	170 (48.4%)	91 (39.4%)
Gender unknown	0 (0.0%)	4 (1.7%)
**Total**	**351 (100.0%)**	**231 (100.0%)**
Age, years		
0–1	10 (2.8%)	13 (5.6%)
>1–5	3 (0.9%)	2 (0.9%)
>5–21	10 (2.8%)	4 (1.7%)
>21–65	142 (40.5%)	90 (39.0%)
>65	181 (51.6%)	107 (46.3%)
Age unknown	5 (1.4%)	15 (6.5%)
**Total**	**351 (100.0%)**	**231 (100.0%)**
Participant status		
Emergency room	68 (19.4%)	2 (0.9%)
Hospitalized	262 (74.6%)	18 (7.8%)
Long-term care	2 (0.6%)	0 (0.0%)
Outpatient	1 (0.3%)	0 (0.0%)
Status unknown	18 (5.1%)	211 (91.3%)
**Total**	**351 (100.0%)**	**231 (100.0%)**
Blood culture bottle type		
BD BACTEC lytic anaerobic	65 (18.5%)	8 (3.5%)
BD BACTEC plus aerobic	61 (17.4%)	20 (8.7%)
BD BACTEC standard aerobic	19 (5.4%)	89 (38.5%)
BD BACTEC standard anaerobic	5 (1.4%)	0 (0.0%)
BacT/ALERT FA plus	72 (20.5%)	29 (12.6%)
BacT/ALERT FN plus	77 (21.9%)	26 (11.3%)
BacT/ALERT PF plus	3 (0.9%)	0 (0.0%)
BacT/ALERT SA standard aerobic	22 (6.3%)	0 (0.0%)
BacT/ALERT SN standard anaerobic	16 (4.6%)	0 (0.0%)
BD BACTEC plus anaerobic	1 (0.3%)	0 (0.0%)
BD BACTEC peds plus	10 (2.8%)	1 (0.4%)
BD BACTEC (unknown aerobic)	0 (0.0%)	5 (2.2%)
BD BACTEC (unknown anaerobic)	0 (0.0%)	3 (1.3%)
BD BACTEC (unknown)	0 (0.0%)	15 (6.5%)
Unknown aerobic	0 (0.0%)	4 (1.7%)
Unknown anaerobic	0 (0.0%)	1 (0.4%)
Bottle type unknown	0 (0.0%)	30 (13.0%)
**Total**	**351 (100.0%)**	**231 (100.0%)**

^*a*^ Bolded rows indicate cumulative totals for each group/parameter.

### Clinical performance

#### Prospective and pre-selected specimens

Of the 381 specimens enrolled in the prospective arm of the study, 30 specimens were excluded from the analysis (*n* = 1 duplicate patient enrollment, and *n* = 29 with incomplete reference testing results due to mixed growth on subculture, insufficient growth, or no growth), leaving a total of 351 prospectively collected specimens for analysis. All 231 pre-selected specimens were included in the analysis. The primary comparator was VITEK 2 for most organisms; BDS was the reference method for *K. variicola*, *A. baumanni*i, *K. oxytoca*, *K. pneumoniae*, and the AMR genes and was also used for resolving discrepancies between the LIAISON PLEX BCN Assay and VITEK 2. Across all 8,687 calls made with clinical specimens, 56.6% (*n* = 4,914/8,687) used VITEK 2 alone, 31.3% (*n* = 2,720/8,687) used BDS alone, and 12.1% (*n* = 1,053/8,687) used both tests as the comparator to the LIASION PLEX BCN Assay.

Clinical performance parameters (sensitivity/PPA, specificity/NPA) of the LIAISON PLEX BCN Assay compared to the reference method for clinical samples are summarized in Table 3. Assay performance was similarly excellent between prospective versus pre-selected specimens, for nearly all gram-negative bacterial targets, as indicated by narrow 95% CIs, demonstrating high precision. The results pooled from prospective and pre-selected samples yielded sensitivities ranging from 90.0% to 100% for 18 of 19 bacterial targets, of which 17 targets were >96%. A notable exception was *Neisseria meningitidis*, for which the LIAISON PLEX BCN Assay showed a sensitivity/PPA of 33% (95% CI: 6.1%‒79.2%) by identifying only one of three positive specimens in the pre-selected cohort (although detected in 50 of 50 contrived specimens comprising six strains). Sensitivity for detecting AMR genes in clinical samples was 97.8%–100%. The LIAISON PLEX BCN Assay also demonstrated similarly excellent specificity/NPA for detecting targets in prospective and pre-selected specimens, at 99.3%–100% for all 19 gram-negative bacterial targets and 98.7%–100% for the eight included AMR genes (Table 3). The overall performance across all targets was 98.6% sensitivity/PPA and 99.9% specificity/NPA.

**TABLE 3:** LIAISON PLEX Gram-Negative Blood Culture Assay performance with prospective and pre-selected specimens^*i*^^,^^*j*^

Target		Sensitivity/PPA			Specificity/NPA		
		TP/(TP + FN)	%	95% CI	TN/(TN + FP)	%	95% CI
Bacteria							
*Acinetobacter baumannii*	Prospective^*a*^	0/0	N/A	N/A	350/350	100%	98.9%–100%
	Pre-selected^*b*^	30/31	96.8%	83.8%–99.4%	199/199	100%	98.1%–100%
	**Combined**	**30/31**	**96.8%**	**83.8%–99.4%**	**549/549**	**100%**	**99.3%–100%**
*Acinetobacter* spp.	Prospective	3/5	60%	23.1%–88.2%	345/345	100%	98.9%–100%
	Pre-selected	33/35	94.3%	81.4%–98.4%	195/195	100%	98.1%–100%
	**Combined**	**36/40** ^*c*^	**90.0%**	**76.9%–96%**	**540/540**	**100%**	**99.3%–100%**
*Citrobacter* spp.	Prospective	6/6	100%	61.0%–100%	344/344	100%	98.9%–100%
	Pre-selected	23/24	95.8%	79.8%–99.3%	206/206	100%	98.2%–100%
	**Combined**	**29/30**	**96.7%**	**83.3%–99.4%**	**550/550**	**100%**	**99.3%–100%**
*Enterobacter* spp.	Prospective	20/20	100%	83.9%–100%	330/330	100%	98.8%–100%
	Pre-selected	9/10	90.0%	59.6%–98.2%	220/220	100%	98.3%–100%
	**Combined**	**29/30**	**96.7%**	**83.3%–99.4%**	**550/550**	**100%**	**99.3%–100%**
*Enterobacteriaceae /Morganellaceae*	Prospective	305/306	99.7%	98.2%–99.9%	44/44	100%	92.0%–100%
	Pre-selected	123/123	100%	97.0%–100%	106/107	99.1%	94.9%–99.8%
	**Combined**	**428/429**	**99.8%**	**98.7%–100%**	**150/151**	**99.3%**	**96.3%–99.9%**
*Escherichia coli*	Prospective	178/179	99.4%	96.9%–99.9%	169/171	98.8%	95.8%–99.7%
	Pre-selected	1/1	100%	20.7%–100%	229/229	100%	98.4%–100%
	**Combined**	**179/180**	**99.4%**	**96.9%–99.9%**	**398/400** ^*d*^	**99.5%**	**98.2%–99.9%**
*Haemophilus influenzae*	Prospective	8/8	100%	67.6%–100%	342/342	100%	98.9%–100%
	Pre-selected	24/24	100%	86.2%–100%	206/206	100%	98.2%–100%
	**Combined**	**32/32**	**100%**	**89.3%–100%**	**548/548**	**100%**	**99.3%–100%**
*Klebsiella oxytoca*	Prospective	9/9	100%	70.1%–100%	340/341	99.7%	98.4%–99.9%
	Pre-selected	26/27	96.3%	81.7%–99.3%	203/203	100%	98.1%–100%
	**Combined**	**35/36**	**97.2%**	**85.8%–99.5%**	**543/544** ^*e*^	**99.8%**	**99.0%–100%**
*Klebsiella pneumoniae*	Prospective	44/45	97.8%	88.4%–99.6%	304/305	99.7%	98.2%–99.9%
	Pre-selected	1/1	100%	20.7%–100%	227/229	99.1%	96.9%–99.8%
	**Combined**	**45/46**	**97.8%**	**88.7%–99.6%**	**531/534** ^*f*^	**99.4%**	**98.4%–99.8%**
*Klebsiella variicola*	Prospective	8/8	100%	67.6%–100%	341/341	100%	98.9%–100%
	Pre-selected	4/4	100%	51.0%–100%	197/197	100%	98.1%–100%
	**Combined**	**12/12**	**100%**	**75.8%–100%**	**538/538**	**100%**	**99.3%–100%**
*Morganella morganii*	Prospective	3/3	100%	43.9%–100%	347/347	100%	98.9%–100%
	Pre-selected	5/5	100%	56.6%–100%	225/225	100%	98.3%–100%
	**Combined**	**8/8**	**100%**	**67.6%–100%**	**572/572**	**100%**	**99.3%–100%**
*Neisseria meningitidis*	Prospective	0/0	N/A	N/A	350/350	100%	98.9%–100%
	Pre-selected	1/3	33.3%	6.1%–79.2%	227/227	100%	98.3%–100%
	**Combined**	**1/3** ^*g*^	**33.3%**	**6.1%–79.2%**	**577/577**	**100%**	**99.3%–100%**
*Proteus* spp.	Prospective	23/23	100%	85.7%–100%	327/327	100%	98.8%–100%
	Pre-selected	14/14	100%	78.5%–100%	216/216	100%	98.3%–100%
	**Combined**	**37/37**	**100%**	**90.6%–100%**	**543/543**	**100%**	**99.3%–100%**
*Pseudomonas aeruginosa*	Prospective	28/29	96.6%	82.8%–99.4%	321/321	100%	98.8%–100%
	Pre-selected	6/6	100%	61.0%–100%	224/224	100%	98.3%–100%
	**Combined**	**34/35**	**97.1%**	**85.5%–99.5%**	**545/545**	**100%**	**99.3%–100%**
*Pseudomonas* spp.	Prospective	29/30	96.7%	83.3%–99.4%	320/320	100%	98.8%–100%
	Pre-selected	7/7	100%	64.6%–100%	223/223	100%	98.3%–100%
	**Combined**	**36/37**	**97.3%**	**86.2%–99.5%**	**543/543**	**100%**	**99.3%–100%**
*Salmonella* spp.	Prospective	1/1	100%	20.7%–100%	349/349	100%	98.9%–100%
	Pre-selected	16/16	100%	80.6%–100%	214/214	100%	98.2%–100%
	**Combined**	**17/17**	**100%**	**81.6%–100%**	**563/563**	**100%**	**99.3%–100%**
*Serratia marcescens*	Prospective	9/9	100%	70.1%–100%	340/341	99.7%	98.4%–99.9%
	Pre-selected	20/20	100%	83.9%–100%	209/210	99.5%	97.4%–99.9%
	**Combined**	**29/29**	**100%**	**88.3%–100%**	**549/551**	**99.6%**	**98.7%–99.9%**
*Stenotrophomonas maltophilia*	Prospective	1/1	100%	20.7%–100%	349/349	100%	98.9%–100%
	Pre-selected	10/10	100%	72.2%–100%	219/220	99.5%	97.5%–99.9%
	**Combined**	**11/11**	**100%**	**74.1%–100%**	**568/569**	**99.8%**	**99.0%–100%**
Resistance Marker Genes^*h*^							
CTX-M (*bla*CTX-M)	Prospective	40/41	97.6%	87.4%–99.6%	295/297	99.3%	97.6%–99.8%
	Pre-selected	5/5	100%	56.6%–100%	169/169	100%	97.8%–100%
	**Combined**	**45/46**	**97.8%**	**88.7%–99.6%**	**464/466**	**99.6%**	**98.4%–99.9%**
IMP (*bla*IMP)	Prospective	0/0	N/A	N/A	338/338	100%	98.9%–100%
	Pre-selected	0/0	N/A	N/A	174/174	100%	97.8%–100%
	**Combined**	**0/0**	**N/A**	**N/A**	**512/512**	**100%**	**99.3%–100%**
KPC (*bla*KPC)	Prospective	1/1	100%	20.7%–100%	336/336	100%	98.9%–100%
	Pre-selected	0/0	N/A	N/A	164/164	100%	97.7%–100%
	**Combined**	**1/1**	**100%**	**20.7%–100%**	**500/500**	**100%**	**99.2%–100%**
NDM (*bla*NDM)	Prospective	0/0	N/A	N/A	338/338	100%	98.9%–100%
	Pre-selected	1/1	100%	20.7%–100%	173/173	100%	97.8%–100%
	**Combined**	**1/1**	**100%**	**20.7%–100%**	**511/511**	**100%**	**99.3%–100%**
OXA (*bla*OXA)	Prospective	0/0	N/A	N/A	332/337	98.5%	96.6%–99.4%
	Pre-selected	26/26	100%	87.1%–100%	137/138	99.3%	96.0%–99.9%
	**Combined**	**26/26**	**100%**	**87.1%–100%**	**469/475**	**98.7%**	**97.3%–99.4%**
VIM (*bla*VIM)	Prospective	0/0	N/A	N/A	338/338	100%	98.9%–100%
	Pre-selected	0/0	N/A	N/A	174/174	100%	97.8%–100%
	**Combined**	**0/0**	**N/A**	**N/A**	**512/512**	**100%**	**99.3%–100%**
MCR	Prospective	0/0	N/A	N/A	334/334	100%	98.9%–100%
	Pre-selected	0/0	N/A	N/A	131/131	100%	97.2%–100%
	**Combined**	**0/0**	**N/A**	**N/A**	**465/465**	**100%**	**99.2%–100%**
SME	Prospective	0/0	N/A	N/A	10/10	100%	72.2%–100%
	Pre-selected	0/0	N/A	N/A	21/21	100%	84.5%–100%
	**Combined**	**0/0**	**N/A**	**N/A**	**31/31**	**100%**	**89.0%–100%**

^*a*^ Of 350/351 prospective specimens with valid results, clinical sites 1–4 accounted for 148, 102, 58, and 42, respectively.

^*b*^ Of 230/231 pre-selected with valid results, clinical sites 1–4 tested 23, 90, 23, and 70 pre-selected specimens, respectively, with the remaining 24 pre-selected specimens tested at Luminex Corp.

^*c*^ Clinical isolates from 2/4 *Acinetobacter* spp. false-negative (FN) specimens were negative for *Acinetobacter* spp. by BDS as no fragment was generated for sequence analysis. One was *A. lwoffii* by VITEK two and *A. berezinia*e by MALDI-TOF MS; the other was *A. lwoffii* by VITEK two and *Moraxella* species by MALDI-TOF MS.

^*d*^ The clinical isolate from 1/2 *E. coli* false-positive (FP) specimens was positive for *E. coli* by BDS.

^*e*^ The *K. oxytoca* FP specimen was positive for *K. oxytoca* by standard of care (both by Verigene BC-GN and Beckman Coulter MicroScan WalkAway automated biochemical identification method).

^*f*^ Two of the three (2/3) *K. pneumoniae* FP specimens were positive for *K. pneumoniae* by standard of care (one by Verigene BC-GN and one by MALDI-TOF MS).

^*g*^ Both *N. meningitidis* FN specimens were positive by another FDA-cleared molecular assay (BioFire FilmArray).

^*h*^ The LIAISON PLEX BCN Assay will report the presence or absence of resistance markers only if an applicable organism is also detected; therefore, the total number of evaluable samples for each resistance marker is dependent on the number of applicable organisms enrolled.

^*i*^ PPA, Positive Percent Agreement; NPA, Negative Percent Agreement; TP, True Positive; TN, True Negative; FP, False Positive; FN, False Negative; CI, Confidence Interval; N/A, Not applicable.

^*j*^ The bold values are combined performance of prospective and pre-selected.

The LIAISON PLEX BCN Assay provided good reliability in pathogen detection within prospective specimens across all four sites, regardless of the blood culture system used (Table 4). Only 8 of the 627 total results were false negative and were only observed for the *Acinetobacter* spp., *Enterobacteriaceae*/*Morganellaceae*, *E. coli*, *K. pneumoniae*, *P. aeruginosa*, *Pseudomonas* spp., and CTX-M targets. Within-site PPA values averaged across pathogens in the prospective group ranged from 98.1%–98.7% and were similar across blood culture systems. Individual pathogen-specific PPAs did differ across sites, but this observation seemed to be largely driven by the presence of occasional false-negative calls in specimens containing low-incidence pathogens (e.g., *Acinetobacter* spp.).

**TABLE 4:** LIAISON PLEX Gram-Negative Blood Culture Assay false-negative results with prospective specimens by site and blood culture system^*a*^

Target	SITE 1			SITE 2			SITE 3			SITE 4			Total		
	BacT/ALERT VIRTUO (*N* = 276/351)			BACTEC (*N* = 182/351)			BACTEC (*N* = 102/351)			BacT/ALERT 3D (*N* = 75/351)					
	TP	FN	PPA	TP	FN	PPA	TP	FN	PPA	TP	FN	PPA	TP	FN	PPA
*Acinetobacter* spp.	1	0	100%	1	1	50.0%	1	0	100%	0	1	0.0%	3	2	60.0%
*Enterobacteriaceae/Morganellaceae*	132	1	99.0%	86	0	100%	50	0	100%	37	0	100%	305	1	99.7%
*E. coli*	71	1	98.6%	49	0	100%	30	0	100%	28	0	100%	178	1	99.4%
*K. pneumoniae*	23	0	100%	12	0	100%	7	1	88.0%	2	0	100%	44	1	97.8%
*P. aeruginosa*	12	0	100%	10	1	91.0%	4	0	100%	2	0	100%	28	1	96.6%
*Pseudomonas* spp.	12	0	100%	11	1	91.7%	4	0	100%	2	0	100%	29	1	96.7%
CTX-M	22	1	95.7%	10	0	100%	5	0	100%	3	0	100%	40	1	97.6%
TOTAL	273	3	98.9%	179	3	98.4%	101	1	99.0%	74	1	98.7%	627	8	98.7%

^*a*^ TP, True Positive; FN, False Negative; PPA, Positive Percent Agreement.

Of the 351 specimens in the prospective group, 330 (94.0%) yielded valid LIAISON PLEX Gram-Negative Blood Culture Assay results (i.e., Detected or Not Detected) on the first attempt. Upon retesting the 21 specimens (6.0%) with an initially invalid result, 20 of 21 specimens generated a valid result after a single retest for a final success rate of 99.7% (*n* = 350/351). Clinical study sites 1–4 accounted for 148, 102, 58, and 42 prospectively collected specimens with valid results, respectively. Of the 231 pre-selected specimens, 226 (97.8%) yielded valid assay results on the first attempt. The five specimens (2.2%) with an initially invalid result were retested, and four of five specimens generated a valid result after a single retest for a final success rate of 99.6% (*n* = 230/231). Clinical study sites 1–4 tested 23, 90, 23, and 70 pre-selected specimens with valid results, respectively, with the remaining 24 pre-selected specimens tested at Luminex Corporation.

The *Enterobacteriaceae/Morganellaceae* families were detected in 87.1% (*n* = 305/350) of the prospectively collected US specimens. At the species/genera level, the three most commonly detected gram-negative bacterial targets in the prospective sample set were *E. coli* at 51.4% (*n* = 180/350), *K. pneumoniae* at 12.9% (*n* = 45/350), and *Pseudomonas* spp. at 8.3% (*n* = 29/350), collectively accounting for 73% of the identified infections (10). The *Pseudomonas* spp. were primarily *P. aeruginosa* (*n* = 28/29) with a single identification (*n* = 1/29) of *P. stutzeri*. The LIAISON PLEX BCN Assay reports the presence or absence of resistance markers only if an associated organism is also detected. The two most commonly detected AMRs were CTX-M (*bla*_CTX-M_), identified in 12.4% (*n* = 42/338) of prospective specimens, and OXA (*bla*_OXA_), detected in 1.5% (*n* = 5/337) of prospective specimens with appropriate positivity for an associated gram-negative bacterial pathogen.

Of the total prospective and pre-selected specimens, the collection sites provided Verigene BC-GN data for 226/582 (38.8%) specimens and MALDI-TOF MS data for 250/582 (43.0%) specimens from standard of care testing. In exploratory analyses that directly compared the performance of the LIAISON PLEX BCN Assay to the eight common bacterial pathogens on the Verigene BC-GN panel, PPAs ranged from 87.5% to 100%, NPAs ranged from 99.0% to 100%, and accuracy ranged from 98.7% to 100%. Discordant findings between LIAISON PLEX and Verigene were then resolved by comparison to the reference comparator result (VITEK 2 + BDS); this approach yielded 100% PPA for six of eight common targets (range 97.1%–100%), 100% NPA for 8 of 8 common targets, and 100% accuracy for 6 of 8 common targets (range 99.6%–100%) (Table S2). Similarly, in an exploratory comparison of LIAISON PLEX BCN Assay results with MALDI-TOF MS findings for 17 bacterial targets, PPAs ranged from 81.5% to 100%, NPAs ranged from 99.2% to 100%, and accuracy ranged from 99.2% to 100%. When discordant findings were resolved according to VITEK 2 results, this yielded 100% PPA for 13 of 16 common targets (range 94.1%–100%), 100% NPA for 15 of 17 common targets (range 99.3%–100%), and 100% accuracy for 11 of 17 common targets (range 99.2%–100%) (Table S3).

#### Contrived specimens

LIAISON PLEX BCN Assay performance was excellent across all assay targets in 746 contrived specimens (Table 5). The lowest sensitivity was 97.2% for *P. aeruginosa*, and the lowest specificity was 99.6% for the *Enterobacteriaceae*/*Morganellaceae* family. In the case of *N. meningitidis*, which showed a sensitivity of 33% in the pre-selected cohort, LIAISON PLEX BCN Assay sensitivity in contrived specimens was 100% (*n* = 50/50 detected comprising 6 unique strains, i.e., ATCC 13077 [serotype A], 13090 [B], 13113 [C], 35561 [Y], and 6250 [unknown], and NTCC 10790 [X]; *n* = 1–11 determinations per strain). Sensitivity was 100% for all eight AMR genes in contrived specimens. Specificity was 99.7% for CTX-M and OXA (oxacillinase; β-lactamase) due to a single false-positive call in each, and 100% for the remaining six AMR genes. Sequencing analysis confirmed that the LIAISON PLEX BCN assay broadly detected multiple OXA family members, including the 1, 9, 10, 23, 24/40, 48, 51, 58, and 143 groups.

**TABLE 5:** LIAISON PLEX Gram-Negative Blood Culture Assay performance with contrived specimens^*a*^^,*c*^

Pathogen/Target Analyte	Sensitivity			Specificity		
	TP/(TP + FN)	%	95% CI	TN/(TN + FP)	%	95% CI
Bacteria						
*A. baumannii*	25/25	100%	86.7%–100%	720/720	100%	99.5%–100%
*Acinetobacter* spp.	45/45	100%	92.1%–100%	700/700	100%	99.5%–100%
*Citrobacter* spp.	32/32	100%	89.3%–100%	713/713	100%	99.5%–100%
*Enterobacter* spp.	54/54	100%	93.4%–100%	691/691	100%	99.4%–100%
*Enterobacteriaceae/Morganellaceae*	460/460	100%	99.2%–100%	284/285	99.6%	98.0%–99.9%
*E. coli*	41/41	100%	91.4%–100%	704/704	100%	99.5%–100%
*H. influenzae*	0/0	0%	0.0%–0.0%	745/745	100%	99.5%–100%
*K. oxytoca*	20/20	100%	83.9%–100%	725/725	100%	99.5%–100%
*K. pneumoniae*	46/46	100%	92.3%–100%	698/699	99.9%	99.2%–100%
*K. variicola*	50/50	100%	92.9%–100%	695/695	100%	99.5%–100%
*M. morganii*	62/62	100%	94.2%–100%	683/683	100%	99.4%–100%
*N. meningitidis*	50/50	100%	92.9%–100%	695/695	100%	99.5%–100%
*Proteus* spp.	30/30	100%	88.6%–100%	715/715	100%	99.5%–100%
*P. aeruginosa*	35/36	97.2%	85.8%–99.5%	709/709	100%	99.5%–100%
*Pseudomonas* spp.	57/58	98.3%	90.9%–99.7%	687/687	100%	99.4%–100%
*Salmonella* spp.	70/70	100%	94.8%–100%	675/675	100%	99.4%–100%
*Serratia marcescens*	55/55	100%	93.5%–100%	690/690	100%	99.4%–100%
*Stenotrophomonas maltophilia*	49/49	100%	92.7%–100%	696/696	100%	99.5%–100%
Resistance Marker Genes^*b*^						
CTX-M (*bla*CTX-M)	105/105	100%	96.5%–100%	305/306	99.7%	98.2%–99.9%
IMP (*bla*IMP)	54/54	100%	93.4%–100%	357/357	100%	98.9%–100%
KPC (*bla*KPC)	61/61	100%	94.1%–100%	350/350	100%	98.9%–100%
NDM (*bla*NDM)	67/67	100%	94.6%–100%	344/344	100%	98.9%–100%
OXA (*bla*OXA)	35/35	100%	90.1%–100%	375/376	99.7%	98.5%–100%
VIM (*bla*VIM)	50/50	100%	92.9%–100%	361/361	100%	98.9%–100%
MCR	50/50	100%	92.9%–100%	326/326	100%	98.8%–100%
SME	50/50	100%	92.9%–100%	5/5	100%	56.6%–100%

^*a*^ Of 745/746 contrived specimens with valid results, clinical sites 1–4 tested 176, 136, 124, and 204 specimens, respectively, with the remaining 105 contrived specimens tested at Luminex Corp.

^*b*^ The LIAISON PLEX BCN Assay will report the presence or absence of resistance markers only if an applicable organism is also detected; therefore, the total number of evaluable samples for each resistance marker is dependent on the number of applicable organisms enrolled.

^*c*^ TP, True Positive; TN, True Negative; FP, False Positive; FN, False Negative; CI, Confidence Interval.

Of the 746 contrived specimens included in the study analysis, 717 specimens (96.1%) generated valid LIAISON PLEX BCN Assay results (i.e., Detected or Not Detected) on the first attempt. The 29 specimens (3.9%) with an initially invalid result were retested, and 28 of 29 specimens generated a valid result after a single retest for a final success rate of 99.9% (*n* = 745/746). Clinical study sites 1–4 tested 176, 136, 124, and 204 contrived specimens with valid results, respectively, with the remaining 105 contrived specimens tested at Luminex Corporation.

Co-infections were identified in 0.6% (*n* = 2/351) of the prospective cohort samples by the LIAISON PLEX BCN Assay. Both co-infections contained two organisms—one specimen was positive for *Enterobacter* spp. + *Enterobacteriaceae*/*Morganellaceae + E*. coli + OXA, and the other was positive for *Enterobacter* spp. + *Enterobacteriaceae*/*Morganellaceae + K. variicola*. The former dual infection (50%; *n* = 1/2) occurred in a 62-year-old man and was concordant with the reference method, and the latter occurred in a 71-year-old man and contained one organism that was not detected by the reference method.

### Target detection

In the Growth and Detection study, the LIAISON PLEX BCN Assay demonstrated 100% positivity for target-positive samples in *n* = 9 of 9 determinations (*n* = 3 bottles × 3 repeats) for each of 14 different gram-negative bacteria, both at initial ring positivity and at ring positivity + 8 h (Table S4). At initial ring positivity, organism concentrations ranged from 1.88 × 10^8^ CFU/mL (*N. meningitidis*) to 2.26 × 10^9^ CFU/mL for *P. mirabilis*. The LIAISON PLEX BCN Assay showed 0% positivity when tested with negative blood culture (*n* = 3/3; not shown).

### Matrix equivalency

The LIAISON PLEX BCN Assay displayed 100% fidelity in accurately identifying representative gram-negative target organisms with resistance gene markers (*n* = 21 targets), with a 0% false-detection rate for 11 off-target gram-positive bacteria and NBM, across 12 different types of commercially available blood culture media bottles from three manufacturers (Table S6). A total of 650 independent blood culture bottles were evaluated, which included 506 gram-negative bottle cultures, 120 gram-positive bottle cultures, and 24 NBM bottles. The average concentration of each organism grown in 12 different types of media bottles is shown in Table S5, representing the approximate concentrations obtained from a mono-bacterial blood culture used in this testing (10^8^–10^9^ CFU/mL). While not included in this evaluation, the bioMérieux BACT/ALERT FA Plus blood culture bottle type was deemed compatible with LIAISON PLEX BCN Assay testing as the standard medium used in the clinical assessment and in other analytical performance tests.

### Analytical reactivity (inclusivity)

The analytical reactivity study demonstrated 100% positivity when the LIAISON PLEX BCN Assay interrogated 246 bacterial strains representing on-panel reportable bacteria, including expected AMR gene positivity for 245 of the 246 on-panel organisms (data not shown). Tested organisms at ring positivity had concentrations ranging from 7.9 × 10^6^ to 3.0 × 10^9^ CFU/mL.

### Analytical specificity (cross-reactivity)

Of the 113 off-panel species tested, 17 species generated positive results and 11 species were cross-reactive (Table 6; Table S7). All cross-reactive species were gram-negative bacteria; no cross-reactivity was observed with the 61 gram-positive bacteria or 11 fungal pathogens tested.

**TABLE 6:** Cross-reactivity of the LIAISON PLEX BCN Assay with off-panel organisms

Organism	Positivity^a^	Organism	Positivity
Gram Positive			
*Abitrophia defective*	0%	*Leuconostoc carnosum*	0%
*Aerococcus viridans*	0%	*Leuconostoc mesenteroides*	0%
*Arcanobacterium bernardiae*	0%	*Listeria grayi*	0%
*Arcanobacterium haemolyticum*	0%	*Listeria innocua*	0%
*Bacillus cereus*	0%	*Listeria ivanovii*	0%
*Bacillus licheniformis*	0%	*Listeria welshimeri*	0%
*Bacillus subtilis*	0%	*Micrococcus luteus*	0%
*Bacillus thuringiensis*	0%	*Micrococcus lylae*	0%
*Corynebacterium amycolatum*	0%	*Pediococcus acidilactici*	0%
*Corynebacterium diphtheriae*	0%	*Pediococcus pentosaceus*	0%
*Corynebacterium pseudodiptheriticum*	0%	*Peptostreptococcus anaerobius*	0%
*Corynebacterium striatum*	0%	*Planococcus citreus*	0%
*Cutibacterium acnes*	0%	*Planococcus kocurii*	0%
*Cutibacterium avidum*	0%	*Rothia dentocariosa*	0%
*Propionibacterium freudenreichii*	0%	*Rothia (Stomatococcus) mucilaginosa*	0%
*Enterococcus avium*	0%	*Staphylococcus capitis*	0%
*Enterococcus casseliflavis,* VRE, *vanC*	0%	*Staphylococcus caprae*	0%
*Enterocococcus dispar*	0%	*Staphylococcus cohnii*	0%
*Enterococcus durans*	0%	*Staphylococcus haemolyticus*	0%
*Enterococcus flavescens*	0%	*Staphylococcus hominis*	0%
*Enterococcus gallinaru*m, *vanC*	0%	*Staphylococcus intermedius*	0%
*Enterococcus hirae*	0%	*Staphylococcus lugdunensis*	0%
*Enterococcus mundtii*	0%	*Streptococcus agalactiae*	0%
*Enterococcus raffinosus*	0%	*Streptococcus anginosus*	0%
*Erysipelothrix rhusiopathiae*	0%	*Streptococcus bovis*	0%
*Kocuria kristinae*	0%	*Streptococcus constellatus*	0%
*Kytococcus sedentarius,* Met R	0%	*Streptococcus equinus*	0%
*Lactobacillus acidophilus*	0%	*Streptococcus mitis*	0%
*Lactobacillus crispatus*	0%	*Streptococcus pneumoniae*	0%
*Lactobacillus rhamnosus*	0%	*Streptococcus pyogenes*	0%
*Corynebacterium frankenforstense*	0%		
Gram Negative			
*Aggregatibacter aphrophilus*	0%	*Fusobacterium nucleatum*	0%
*Bacteroides fragilis*	0%	*Haemophilus haemolyticus*	0%
*Brevundimonas diminuta*	0%	*Herbaspirillum huttiense*	0%
*Burkholderia cepacian*	0%	*Kingella kingae*	0%
*Capnocytophaga ochracea*	0%	*Moraxella catarrhalis*	0%
*Cardiobacterium hominis*	0%	*Neisseria lactamica*	0%
*Comamonas testosterone*	0%	*Neisseria mucosa*	0%
*Delftia acidovorans*	0%	*Neisseria sicca*	0%
*Eikenella corrodens*	0%	*Parabacteroides distasonis*	0%
*Elizabethkingia meningoseptica*	0%	*Pasteurella aerogenes*	0%
*Fusobacterium necrophorum*	0%	*Prevotella bivia*	0%
*Aeromonas hydrophila*	0%	*Klebsiella quasivariicola*	100%
*Haemophilus parainfluenzae*	0%	*Providencia rustigiani*	100%
*Haemophilus parahaemolyticus*	33%	*Providencia vermicola*	100%
*Enterobacter kobei*	100%	*Pseudomonas paraeruginosa*	100%
*Haemophilus aegyptius*	100%	*Serratia entomophila*	100%
*Klebsiella grimontii*	100%	*Serratia nevei*	100%
*Klebsiella huaxiensis*	100%	*Serratia odorifera*	100%
*Klebsiella michiganensis*	100%	*Serratia quinivorans*	100%
*Klebsiella quasipneumoniae*	100%	*Serratia ureilytica*	100%
*Lelliottia amnigena*	100%		
Yeast/Fungi			
*Aspergillus fumigatus*	0%	*Candida tropicalis*	0%
*Candida albicans*	0%	*Cryptococcus neoformans*	0%
*Candida famata*	0%	*Kluyveromyces lactis*	0%
*Candida glabrata*	0%	*Saccharomyces cerevisiae*	0%
*Candida krusei*	0%	*Schizosaccharomyces pombe*	0%
*Candida parapsilosis*	0%		

^a^ Each of the 113 off-panel organisms was grown in blood culture bottles and tested in triplicate assay cartridges.

### Interfering substances

All four representative on-panel targets (*A. baumannii*, *E. coli*, *H. influenzae*, and *N. meningitidis*) demonstrated 100% positivity in the presence of each of six potential interfering substances (unconjugated bilirubin 20 mg/dL, conjugated bilirubin 20 mg/dL, hemoglobin 14 g/L, intralipid 3 g/dL, γ-globulin 6 g/dL, and sodium polyanethosulfonate 0.25% wt/vol), and 0% target detection was observed in the negative control sample in the presence of the same interferents (five replicates each; data not shown). Additionally, 100% on-panel target detection was observed for the positive control for each target.

### Competitive inhibition/co-infection and microbial interference

In pairwise competitive inhibition/co-infection experiments using 10 on-panel bacteria, a low-concentration of each on-panel target was accurately identified 100% of the time by the LIAISON PLEX BCN Assay in the presence of high concentrations of each of the other nine on-panel targets (Table S8).

In microbial interference studies, low concentrations of the same 10 representative on-panel bacteria were accurately detected in the presence of high concentrations of seven off-panel, clinically relevant, gram-positive potential bacterial interferents (Table S9).

### Multi-site and interoperator reproducibility

Overall percent agreement across a total of six operators at three sites was 99.9% for both positive and negative samples (*n* = 1,440/1,441 total data points) when tested by the LIAISON PLEX BCN Assay (additional details in Reference 10). Agreement was 100% for all on-panel calls (both concentrations) and the NBM. The sole incongruity was a single erroneous *Enterobacteriaceae*/*Morganellaceae* call for a *S. aureus*-negative control sample by one operator at one site. In a related within-laboratory inter-operator repeatability study of the LIAISON PLEX BCN Assay, call agreement between two operators was 99.8% (10).

## DISCUSSION

Timely initiation of targeted antibiotic therapy is essential for achieving optimal outcomes when treating bacteremia; every hour that effective antibiotic therapy is delayed in septic shock decreases survival by 7%‒8% (4). The LIAISON PLEX Gram-Negative Blood Culture Assay reliably and rapidly identified 19 targeted bacterial pathogens and simultaneously determined the presence or absence of 8 clinically important AMRs in positive blood cultures from patients with BSI. The LIAISON PLEX BCN Assay panel identifies all three of the gram-negative bacterial groups designated by the WHO as “critical priority” (i.e., third-generation cephalosporin-resistant Enterobacterales, carbapenem-resistant Enterobacterales, and carbapenem-resistant *A. baumannii*) and several pathogens deemed “high priority” (8). The LIAISON PLEX BCN Assay also detects AMR infections identified as urgent public health threats in the U.S. Centers for Disease Control’s 2024 Antimicrobial Resistance Threats update, that is, increased cases of hospital-acquired Enterobacterales (especially *E. coli* and *K. pneumonia*) and *A. baumannii* (9). Sample-to-answer time with the LIAISON PLEX BCN Assay was approximately 2 h, which is significantly shorter than the sometimes days required for follow-up species typing and susceptibility determinations by subculture methods or the 9–18 h typically required by automated biochemical detection and susceptibility testing platforms such as VITEK 2 (5, 14).

Clinical performance results (prospective + pre-selected specimens) indicated high sensitivity/PPA with the LIAISON PLEX BCN Assay for nearly all bacterial targets, that is, >96% for 17 of 19 targets. Two of four *Acinetobacter* spp. false-negative specimens were negative for *Acinetobacter* spp. by BDS. One isolate was identified as *A. lwoffii* by VITEK 2 and *A. bereziniae* by MALDI-TOF MS, while the other was identified as *A. lwoffii* by VITEK 2 and as a *Moraxella* species by MALDI-TOF MS. *A. lwoffii* exhibits limited and variable biochemical reactivity, which can produce profiles overlapping with other non-fermenting gram-negative organisms such as *Moraxella* and discordant identification by VITEK 2. At the species level, the LIAISON PLEX BCN Assay identified *A. baumannii* in clinical samples with a sensitivity of 96.8%.

The other exception, *N. meningitidis*, was identified in only one of three positive specimens in the pre-selected cohort (PPA = 33%). The two samples with missed detections were collected from the same hospital system, from different patients, approximately 4 months apart. No *N. meningitidis* occurred in the prospective cohort. All three pre-selected samples had been identified as *N. meningitidis*-positive based on standard of care results provided by the vendor and were confirmed in-house as positive using the VITEK 2 system. Thus, these missed detections were unexpected, especially because the LIAISON PLEX BCN Assay correctly identified six different strains of *N. meningitidis* in 50/50 contrived specimens. Additionally, analytical studies of inclusivity utilizing wet testing and *in silico* analysis did not identify any issue in detecting the interrogated strains of *N. meningitidis*. Regrowth/retesting of the three frozen banked specimens again yielded one of three *N. meningitidis*-positive versus three of three positive by another FDA-cleared molecular assay.

To assess potential sequence mismatches in the oligo-binding sites used by the LIAISON PLEX BCN Assay, we performed BDS analysis with primers that amplified the target region. In the one pre-selected sample detected by the BCN Assay, the assay oligos were a perfect match to the target region; however, in the two undetected specimens, we identified a 676 base-pair deletion encompassing the assay target region. This deletion was not present in any of the 129 complete genome sequences for *N. meningitidis* in the National Center for Biotechnology Information database and encompassed the assay target sequence. Performing BDS on six additional genes/regions confirmed that the missed specimens were indeed *N. meningitidis*. Thus, while the LIAISON PLEX BCN Assay was unable to detect this particular deletion-containing *N. meningitidis* strain, its absence from the NCBI database suggests that it is an extremely rare occurrence and that LIAISON PLEX BCN Assay performance should be assessed in the context of all specimens tested.

Sensitivity for detecting target AMR genes in clinical samples, when present, was ≥ 97.8%. The most common AMR gene was CTX-M, detected in 12.4% of clinical specimens; this rate approximates the 11% CTX-M positivity rate reported in 4,209 gram-negative blood cultures from patients across 66 US medical centers (15). Although several AMR gene assay targets were not present in clinical specimens, the LIAISON PLEX BCN Assay detected all eight AMR gene targets with 100% sensitivity in contrived specimens.

Specificity/NPA of the LIAISON PLEX BCN Assay was similarly excellent in prospective and pre-selected specimens. Specificity was ≥ 99.3% for all 19 gram-negative bacterial targets, and ≥ 99.6% for the 8 included AMR genes.

Directly comparing LIAISON PLEX BCN results with MALDI-TOF MS findings for 17 bacterial targets in prospective + pre-selected specimens yielded PPAs ranging from 94.1% to 100%, NPAs ranging from 99.3% to 100%, and an average accuracy of 99.7%. Resolving discordant results according to the VITEK 2 + BDS comparator method yielded 100% PPA for 13 of 16 common targets, 100% NPA for 15 of 17 common targets, and 99.9% average accuracy.

The DNA array-based LIAISON PLEX BCN Assay’s combined 27-target panel represents an expansion versus the predecessor Verigene Gram-Negative Blood Culture Nucleic Acid Test’s combined 14 targets and includes multiple targets not present in the FilmArray and ePlex PCR-based multiplex assays (e.g., *K. variicola*, *Acinetobacter* spp., *Pseudomonas* spp., and SME). *K. variicola* is an emerging pathogen of the *K. pneumoniae* complex that is not currently discernible from other members by automated biochemical testing (14). Indeed, of the 12 *K. variicola* infections detected by the LIAISON PLEX BCN Assay in prospective and pre-selected specimens (all confirmed by BDS), 10 were characterized by VITEK 2 as *K. pneumoniae* subsp. *pneumoniae*, and 2 as *K. oxytoca*. Of those 12 *K. variicola* infections detected by the LIAISON PLEX BCN Assay in clinical specimens, eight had comparative MALDI-TOF MS data provided by the clinical sites that identified six as *K. variicola* and two as *K. pneumoniae*. As hypervirulent AMR strains of *K. variicola* that cause high-mortality BSI continue to emerge (16), differentiation from other complex members becomes increasingly important. The LIAISON PLEX BCN Assay also interrogates SME (*Serratia marcescens* enzyme, *sme* gene), carbapenemases originating from *S. marcescens* that have been increasingly reported in BSI samples from the Americas and the UK (17). This AMR gene target is neither present in the Verigene, FilmArray, nor ePlex assay panels. The LIAISON PLEX BCN Assay reliably characterized the presence of all eight AMR gene targets, including broad coverage of established and emerging OXA β-lactamase family members (18).

Representative gram-negative pathogen targets variably present in the FilmArray and/or ePlex target panels, but not in the LIAISON PLEX BCN Assay, include *B. fragilis*, *F. nucleatum*, *K. aerogenes*, and *P. mirabilis*. Bacteremia due to anaerobic *B. fragilis*, a normal component of the colon microbiota, is an infrequent occurrence generally associated with disruption of the intestinal mucosa by trauma, surgery, or cancer (19). A 2023 Spanish report spanning 13 years indicated that anaerobic bacteria constituted 6.2% of culture-proven sepsis cases (*n* = 491/7,956), of which *B. fragilis* constituted 23.8%, cumulatively accounting for < 2% of BSI cases (20). The incidence of BSI from *F. nucleatum*, an anaerobic oropharyngeal and gastrointestinal commensal bacterium, is even lower (0.22–0.34 cases per 100,000 population) (21). Although *K. aerogenes* (formerly *E. aerogenes*), an important contributor to hospital-acquired BSI (22), is not specifically interrogated by the LIAISON PLEX BCN Assay, it should be captured as both an *Enterobacter* genus and an *Enterobacteriaceae*/*Morganellaceae* family member. Indeed, all six of the prospective specimens that were identified as *K. aerogenes* by VITEK 2 were also identified as *Enterobacter* spp. by the LIASION PLEX BCN Assay (10). Similarly, *P. mirabilis*, typically associated with urinary tract infection and causative for approximately 5%–18% of bacteremia cases in hospitalized elderly (23), will be captured by the LIAISON PLEX BCN Assay as both a *Proteus* genus member and an *Enterobacteriaceae*/*Morganellaceae* family member.

Globally, members of the *Enterobacteriaceae* family are the causative organisms responsible for most gram-negative BSIs (1, 8). Our study identified *Enterobacteriaceae/Morganellaceae* family members in 87.1% of gram-negative BSIs in prospectively collected US specimens, of which *E. coli* and *K. pneumoniae* were respectively responsible for 51% and 13% of infections. Similarly, in a 2023 review of the epidemiology of gram-negative BSI in the US that included data from 24 hospitals, approximately 76% were caused by *Enterobacteriaceae,* with 51% due to *E. coli* and 17% due to *K. pneumoniae* (24). A 2024 Korean nationwide study identified *E. coli* and *K. pneumoniae* as respectively responsible for 37% and 19% of all monomicrobial BSIs—both gram-positive and gram-negative combined—and one or the other was involved in > 80% of all polymicrobial BSIs (25).

Analytical performance of the LIAISON PLEX BCN Assay was excellent, with good assay inclusivity demonstrated by the reliable detection of multiple strains of each bacterial target, compatibility with 13 common commercially available culture bottle/matrix combinations, minimal cross-reactivity with off-panel organisms, and reliable detection of target bacteria in the presence of high concentrations of potentially interfering substances and microorganisms, and in co-infection scenarios. Of the 11 gram-negative off-panel bacteria that cross-reacted with assay targets, only three had a history as infrequently encountered human pathogens. *Klebsiella grimontii*, which was detected as *K. oxytoca*, is one of the nine species encompassing the *K. oyxtoca* complex that are difficult-to-impossible to differentiate based on phenotypic criteria (26). *K. grimontii* isolates have rarely been identified in blood cultures from patients with bacteremia in Western Europe and South Africa (27). *K. quasipneumoniae*, which was detected as closely related and similarly virulent *K. pneumoniae*, has been occasionally isolated from blood specimens in Asia, the US, and Europe, with some strains carrying the *K. pneumoniae* carbapenemase (KPC) AMR gene (28). Finally, *Haemophilus parahaemolyticus*, identified in one of three test bottles as *Enterobacteriaceae/Morganellaceae*, is reportedly a rare cause of septic shock after aspiration pneumonia (29).

In analytical assessments comprising 1,328 samples, the initial success rate was 94.0%, 97.8%, and 96.1% for prospective, pre-selected, and contrived specimens, respectively, giving a cumulative 95.9% of specimens producing valid results on the first evaluation. The final success rate was 99.8% after re-testing initially inconclusive samples with a new assay cartridge. Inter-site and inter-operator reproducibility consistently approached 100%, demonstrating uniform performance of the assay system across different laboratories and personnel.

The LIAISON PLEX BCN Assay is indicated for use alongside other clinical and laboratory findings to assist in diagnosing gram-negative bacterial BSI, and not as a standalone diagnostic approach. The assay rapidly confirms the presence of a combined 27 pathogenic bacterial targets and 8 associated AMR genes that are commonly encountered in positive continuous blood cultures. While the LIAISON PLEX BCN Assay accurately identified multiple sequence variants of each of 19 bacterial targets, there remains the possibility of false-negative calls with untested strains. Qualitative LIAISON PLEX BCN Assay performance may be impacted by concurrent/recent antimicrobial treatment or if circulating target levels are below assay detection limits. While the LIAISON PLEX BCN Assay produced expected results with two common pediatric culture bottles (150 cumulative determinations), it has not been specifically validated in a pediatric population. Strengths of this study included the multicenter prospective design that tested clinical samples from patients of diverse age with a balanced gender ratio, and its detailed analytical performance assessment.

The multiplex LIAISON PLEX BCN Assay demonstrated excellent performance for simultaneously evaluating 19 known gram-negative bacterial pathogens and 8 associated AMR genes in positive blood culture specimens. Although culture-based techniques and biochemical assessment remain essential primary diagnostic approaches for diagnosing bloodstream bacterial infection, the LIAISON PLEX BCN Assay provides a reliable adjunctive approach to quickly confirm the presence of a diverse panel of clinically relevant gram-negative pathogens and AMRs in blood culture.

## Data Availability

The data supporting the findings of this study are available from the corresponding author upon reasonable request.

## SUPPLEMENTAL MATERIAL

Supplemental tables jcm.00018-26-s0001.pdf Tables S1 to S9.

ASM does not own the copyrights to Supplemental Material that may be linked to, or accessed through, an article. The authors have granted ASM a non-exclusive, world-wide license to publish the Supplemental Material files. Please contact the corresponding author directly for reuse.
