# Supplementary material for: Performance of the LIAISON PLEX gram-negative blood culture assay for identifying bacterial pathogens and resistance genes in blood cultures
Source: J Clin Microbiol. 2026 May 22;64(6):e00018-26. doi: 10.1128/jcm.00018-26 (PMC13251429; doi:10.1128/jcm.00018-26)
Supplement: Supplemental tables — Tables S1 to S9. [file jcm.00018-26-s0001.pdf]

LIAISON PLEX® Gram-Negative Blood Culture Assay SUPPLEMENTAL TABLES

Supplemental Table S1. LIAISON PLEX® Gram-Negative Blood Culture Assay results interpretation

Detected Organism or Resistance Marker	Reported Targets			
	Family	Genus[a, b]	Species[a]	Resistance Marker[c, d, e]
Citrobacter spp.	*Enterobacteriaceae / Morganellaceae*	*Citrobacter* spp.	-	-
Enterobacter spp.	*Enterobacteriaceae / Morganellaceae*	*Enterobacter* spp.[f]	-	-
Proteus spp.	*Enterobacteriaceae / Morganellaceae*	*Proteus* spp.	-	-
Salmonella spp.	*Enterobacteriaceae / Morganellaceae*	*Salmonella* spp.	-	-
Escherichia coli	*Enterobacteriaceae / Morganellaceae*	-	*Escherichia coli*[g]	-
Klebsiella oxytoca	*Enterobacteriaceae / Morganellaceae*	-	*Klebsiella oxytoca*	-
Klebsiella pneumoniae	*Enterobacteriaceae / Morganellaceae*	-	*Klebsiella pneumoniae*	-
Klebsiella variicola	*Enterobacteriaceae / Morganellaceae*	-	*Klebsiella variicola*	-
Morganella morganii	*Enterobacteriaceae / Morganellaceae*	-	*Morganella morganii*	-
Serratia marcescens	*Enterobacteriaceae / Morganellaceae*	-	*Serratia marcescens*	-
Acinetobacter spp.	-	*Acinetobacter* spp.	-	-
Acinetobacter baumannii	-	*Acinetobacter* spp.	*Acinetobacter baumannii*	-
Pseudomonas spp.	-	*Pseudomonas* spp.	-	-
Pseudomonas aeruginosa	-	*Pseudomonas* spp.	*Pseudomonas aeruginosa*	-
Haemophilus influenzae	-	-	*Haemophilus influenzae*	-
Neisseria meningitidis	-	-	*Neisseria meningitidis*	-
Stenotrophomonas maltophilia	-	-	*Stenotrophomonas maltophilia*	-
	Enterobacteriaceae / Morganellaceae[h]	-	-	-
CTX-M<br>IMP	Any *Citrobacter* spp., *Enterobacter* spp., *Enterobacteriaceae/Morganellaceae*, *E. coli*, *K. oxytoca*, *K. pneumoniae*, *K. variicola*, *M. morganii*, *Proteus* spp., *Salmonella* spp., *S. marcescens*, *Acinetobacter* spp., *Pseudomonas* spp., *S. maltophilia*			CTX-M<br>IMP

NDM<br>VIM		NDM<br>VIM
KPC<br>OXA	Any *Citrobacter* spp., *Enterobacter* spp., *Enterobacteriaceae/Morganellaceae*, *E. coli*, *K. oxytoca*, *K. pneumoniae*, *K. variicola*, *M. morganii*, *Proteus* spp., *Salmonella* spp., *S. marcescens*, *Acinetobacter* spp., *Pseudomonas* spp.	KPC<br>OXA
MCR	Any *Citrobacter* spp., *Enterobacter* spp., *Enterobacteriaceae/Morganellaceae, E. coli, K. oxytoca, K. pneumoniae, K. variicola, M. morganii, Proteus* spp., *Salmonella* spp., *S. marcescens, Pseudomonas* spp.	MCR
SME	*Serratia marcescens*	SME

[a]Detection of a genus with a corresponding species call does not rule out a coinfection with multiple species within the same genus.

[b]In rare cases, a valid detection of a species may be reported without the corresponding genus detection.

[c]Resistance Marker is reported as "Not Reviewed" if associated organism(s) is not detected.

[d]Negative results for antimicrobial resistance genes do not indicate bacterial susceptibility, as there are multiple mechanisms that can contribute to resistance.

[e]All findings should be used in conjunction with standard of care culture workflows. Therapeutic strategies that include antimicrobial agents with limited or no activity against bacteria producing metallo-beta-lactamases, should be used with caution when resistance markers are detected. Subsequent culture and antimicrobial susceptibility confirmation may be helpful in determining a therapeutic strategy in patients with known or suspected carbapenem-non-susceptible bacterial infections.

[f]*Klebsiella aerogenes* will be reported *Enterobacter* spp.

[g]LIAISON PLEX BCN Assay will not distinguish between *Escherichia coli* and *Shigella* spp. (*S. dysenteriae, S. boydii, S. flexneri* and *S. sonnei*).

[h]Detection of the *Enterobacteriaceae / Morganellaceae* family target alone in the absence of a genus or species indicates that the LIAISON PLEX BCN Assay detected the gene target for *Enterobacteriaceae / Morganellaceae*, but the genus or species could not be differentiated. Additional testing is necessary for species-level identification.

Supplemental Table S2. Exploratory performance of LIAISON PLEX® BCN Assay compared to VERIGENE® BC-GN Standard of Care results for Prospective and Pre-selected samples[a,b]

A) UNRESOLVED, LIAISON PLEX BCN vs Verigene BC-GN Results

Target	TP/TP+FN	PPA[c] %	95% CI	TN/TN+FP	NPA[d] %	95% CI	Accuracy[e] %	95% CI
Acinetobacter spp.	2/2	100%	34.2–100	224/224	100%	98.3–100	100%	98.3–100
Citrobacter spp.	3/3	100%	43.9–100	223/223	100%	98.3–100	100%	98.3–100
Enterobacter spp.	12/14	85.7%	60.1–96.0	212/212	100%	98.2–100	99.1%	96.8–99.8
Escherichia coli	120/122	98.4%	94.2–99.5	103/104	99.0%	94.8–99.8	98.7%	96.2–99.5
Klebsiella oxytoca	8/9	88.9%	56.5–98.0	217/217	100%	98.3–100	99.6%	97.5–99.9
Klebsiella pneumoniae	33/36	91.7%	78.2–97.1	190/190	100%	98.0–100	98.7%	96.2–99.5
Proteus spp.	16/16	100%	80.6–100	210/210	100%	98.2–100	100%	98.3–100
Pseudomonas aeruginosa	16/16	100%	80.6–100	209/210	99.5%	97.4–99.9	99.6%	97.5–99.9

B) RESOLVED, Agreement of 2 of 3 tests—LIAISON PLEX BCN and/or Verigene BC-GN and/or Vitek 2/BDS

Target	TP/TP+FN	PPA[c] %	95% CI	TN/TN+FP	NPA[d] %	95% CI	Accuracy[e] %	95% CI
Acinetobacter spp.	2/2	100%	34.2–100	224/224	100%	98.3–100	100%	98.3–100
Citrobacter spp.	3/3	100%	43.9–100	223/223	100%	98.3–100	100%	98.3–100
Enterobacter spp.	12/12	100%	75.8–100	214/214	100%	98.2–100	100%	98.3–100
Escherichia coli	121/122	99.2%	95.5–99.9	104/104	100%	94.8–99.8	99.6%	96.8–99.8
Klebsiella oxytoca	8/8	100%	67.6–100	218/218	100%	98.3–100	100%	98.3–100
Klebsiella pneumoniae	33/34	97.1%	85.1–99.5	192/192	100%	98.0–100	99.6%	97.5–99.9
Proteus spp.	16/16	100%	80.6–100	210/210	100%	98.2–100	100%	98.3–100
Pseudomonas aeruginosa	17/17	100%	81.6–100	209/209	100%	98.2–100	100%	98.3–100

[a]Comparison of common targets

[b]Discordant results adjudicated by comparison to final clinical comparator result (VITEK 2 and/or BDS).

[c]PPA defined as TP divided by TP+FN

[d]NPA defined as TN divided by TN+FP

[e]Accuracy defined as the percentage of true calls (TP+TN) divided by total calls (TP+TN+FP+FN).

Abbreviations: PPA, Positive Percent Agreement; NPA, Negative Percent Agreement; CI, Confidence Interval.

Supplemental Table S3. Exploratory performance of LIASION PLEX BCN Assay compared to MALDI-TOF MS Standard of Care results for Prospective and Pre-selected samples[a,b]

A) UNRESOLVED, LIAISON PLEX BCN vs MALDI-TOF MS Results

Target	TP/TP+FN	PPA[c] %	95% CI	TN/TN+FP	NPA[d] %	95% CI	Accuracy[e] %	95% CI
Acinetobacter baumannii	16/17	94.1%	73.0–99.0	233/233	100%	98.4–100	99.6%	97.8–99.9
Acinetobacter spp.	3/3	100%	43.9–100	247/247	100%	98.5–100	100%	98.5–100
*Citrobact*er spp.	6/7	85.7%	48.7–97.4	243/243	100%	98.4–100	99.6%	97.8–99.9
Enterobacter spp.	16/16	100%	80.6–100	234/234	100%	98.4–100	100%	98.5–100
Enterobacteriaceae/Morganellaceae	200/202	99.0%	96.5–99.7	48/48	100%	92.6–100	99.2%	97.1–99.8
Escherichia coli	109/109	100%	96.6–100	140/141	99.3%	96.1–99.9	99.6%	97.8–99.9
Haemophilis influenzae	8/8	100%	67.6–100	242/242	100%	98.4–100	100%	98.5–100
Klebsiella pneumoniae	22/27	81.5%	63.3–91.8	223/223	100%	98.3–100	98.0%	95.4–99.1
Klebsiella oxytoca	3/3	100%	43.9–100	247/247	100%	98.5–100	100%	98.5–100
Klebsiella variicola	6/6	100%	61.0–100	242/244	99.2%	97.1–99.8	99.2%	97.1–99.8
Morganella morganii	3/3	100%	43.9–100	247/247	100%	98.5–100	100%	98.5–100
Proteus spp.	21/21	100%	84.5–100	229/229	100%	98.4–100	100%	98.5–100
Pseudomonas aeruginosa	17/17	100%	81.6–100	233/233	100%	98.4–100	100%	98.5–100
Pseudomonas spp.	18/18	100%	82.4–100	232/232	100%	96.9–99.8	100%	98.5–100
Salmonella spp.	1/1	100%	20.7–100	249/249	100%	98.5–100	100%	98.5–100
Serratia marcescens	11/11	100%	74.1–100	238/239	99.6%	98.4–100	99.6%	97.8–99.9
Stenotrophomonas maltophilia	0	N/A[f]		250/250	100%	98.4–100	N/A	

B) RESOLVED, Agreement of 2 of 3 tests—LIAISON PLEX BCN and/or MALDI-TOF MS and/or Vitek 2/BDS

Target	TP/TP+FN	PPA[c] %	95% CI	TN/TN+FP	NPA[d] %	95% CI	Accuracy[e] %	95% CI
Acinetobacter baumannii	16	94.1%	73.0–99.0	233/233	100%	98.4–100	99.6%	97.8–99.9
Acinetobacter spp.	3/3	100%	43.9–100	247/247	100%	98.5–100	100%	98.5–100
Citrobacter spp.	6/6	100%	61.0–100	244/244	100%	98.5–100	100%	98.5–100
Enterobacter spp.	16/16	100%	80.6–100	234/234	100%	98.4–100	100%	98.5–100
Enterobacteriaceae/Morganellaceae	199/200	99.5%	97.2–99.9	50/50	100%	92.7–100	99.2%	97.1–99.8
Escherichia coli	109/109	100%	96.6–100	140/141	99.3%	96.1–99.9	99.6%	97.8–99.9
Haemophilus influenzae	8/8	100%	67.6–100	242/242	100%	98.4–100	100%	98.5–100
Klebsiella pneumoniae	22/22	95.7%	79.0–99.2	227/227	100%	98.3–100	99.6%	97.8–99.9
Klebsiella oxytoca	3/3	100%	43.9–100	247/247	100%	98.5–100	100%	98.5–100
Klebsiella variicola	8/8	100%	67.6–100	242/242	100%	98.4–100	100%	98.5–100
Morganella morganii	3/3	100%	43.9–100	247/247	100%	98.5–100	100%	98.5–100
Proteus spp.	21/21	100%	84.5–100	229/229	100%	98.4–100	100%	98.5–100
Pseudomonas aeruginosa	17/17	100%	81.6–100	233/223	100%	98.4–100	100%	98.5–100
Pseudomonas spp.	18/18	100%	82.4–100	232/232	100%	98.4–100	100%	98.5–100
Salmonella spp.	1/1	100%	20.7–100	249/249	100%	98.5–100	100%	98.5–100
Serratia marcescens	11/11	100%	74.1–100	238/239	99.6%	97.7–99.9	99.6%	97.8–99.9
Stenotrophomonas maltophilia	0	N/A[f]		250/250	100%	98.5–100	N/A	

[a]Comparison of common targets

[b]Discordant results adjudicated by comparison to final clinical comparator result (VITEK 2 and/or BDS)

[c]PPA defined as TP divided by TP+FN

[d]NPA defined as TN divided by TN+FP

[e]Accuracy defined as the percentage of true calls (TP+TN) divided by total calls (TP+TN+FP+FN).

[f]Incomplete MALDI-TOF MS results for this target

Abbreviations: PPA, Positive Percent Agreement; NPA, Negative Percent Agreement; CI, Confidence Interval.

Supplemental Table S4. LIAISON PLEX® Gram-Negative Blood Culture Assay growth & detection results

		Positive Samples					
		Ring-Positive			Ring-Positive + 8 Hours		
**Species Tested**	**Targets Tested**	**Per Bottle (CFU/mL)**	**Mean (CFU/mL)**	**Positive Agreement/ Total (% Detected)**	**Per Bottle (CFU/mL)**	**Mean (CFU/mL)**	**Positive Agreement/ Total (% Detected)**
Acinetobacter baumannii [OXA] IHMA 128307	*Acinetobacter baumannii* | *Acinetobacter* spp. | OXA	2.13×10^{8}<br>2.18×10^{8}<br>1.86×10^{8}	2.06×10^{8}	9/9 (100%)	2.09×10^{8}<br>1.06×10^{8}<br>1.75×10^{8}	1.63×10^{8}	9/9 (100%)
Citrobacter freundii [VIM] IHMA 549813	*Citrobacter* spp. | *Enterobacteriaceae / Morganellaceae* | VIM	9.63×10^{8}<br>1.49×10^{9}<br>1.39×10^{9}	1.28×10^{9}	9/9 (100%)	1.76×10^{9}<br>2.16×10^{9}<br>1.68×10^{9}	1.87×10^{9}	9/9 (100%)
Enterobacter cloacae ATCC 35030	*Enterobacter* spp. | *Enterobacteriaceae / Morganellaceae*	1.46×10^{9}<br>1.61×10^{9}<br>1.68×10^{9}	1.58×10^{9}	9/9 (100%)	1.68×10^{9}<br>2.07×10^{9}<br>2.36×10^{9}	2.04×10^{9}	9/9 (100%)
Escherichia coli [MCR] NCTC 13846	*Enterobacteriaceae / Morganellaceae* | *Escherichia coli* | MCR	1.70×10^{9}<br>1.96×10^{9}<br>1.95×10^{9}	1.87×10^{9}	9/9 (100%)	1.45×10^{9}<br>1.83×10^{9}<br>1.66×10^{9}	1.65×10^{9}	9/9 (100%)
Klebsiella oxytoca [CTX-M] IHMA 683079	CTX-M | *Enterobacteriaceae / Morganellaceae* | *Klebsiella oxytoca*	4.53×10^{8}<br>1.48×10^{9}<br>9.73×10^{8}	9.69×10^{8}	9/9 (100%)	5.3×10^{8}<br>2.9×10^{8}<br>2.04×10^{8}	3.41×10^{8}	9/9 (100%)
Klebsiella pneumoniae [KPC] IHMA 629630	*Enterobacteriaceae / Morganellaceae* | KPC | *Klebsiella pneumoniae*	1.33×10^{9}<br>1.66×10^{9}<br>1.69×10^{9}	1.56×10^{9}	9/9 (100%)	1.55×10^{9}<br>1.61×10^{9}<br>1.59×10^{9}	1.58×10^{9}	9/9 (100%)
Klebsiella variicola ATCC BAA-830	*Enterobacteriaceae / Morganellaceae* | *Klebsiella variicola*	1.66×10^{9}<br>1.82×10^{9}<br>1.78×10^{9}	1.75×10^{9}	9/9 (100%)	2.00×10^{9}<br>2.13×10^{9}<br>2.05×10^{9}	2.06×10^{9}	9/9 (100%)
Salmonella enterica subsp. *enterica* serovar Enteritidis ATCC 13076	*Enterobacteriaceae / Morganellaceae* | *Salmonella* spp.	4.90×10^{7}<br>1.38×10^{8}<br>5.80×10^{8}	2.56×10^{8}	9/9 (100%)	1.88×10^{9}<br>2.06×10^{9}<br>1.61×10^{9}	1.85×10^{9}	9/9 (100%)
Serratia marcescens [SME] NCTC 13920	*Enterobacteriaceae / Morganellaceae* | SME | *Serratia marcescens*	1.55×10^{8}<br>3.27×10^{8}<br>2.32×10^{8}	2.38×10^{8}	9/9 (100%)	1.82×10^{9}<br>1.39×10^{9}<br>1.19×10^{9}	1.47×10^{9}	12/12[a] (100%)

Morganella morganii [NDM] IHMA 605873	*Enterobacteriaceae / Morganellaceae* | *Morganella morganii* | NDM	1.77×10^{9}<br>1.76×10^{9}<br>1.52×10^{9}	1.68×10^{9}	9/9 (100%)	2.75×10^{9}<br>2.65×10^{9}<br>2.74×10^{9}	2.71×10^{9}	9/9 (100%)
Pseudomonas aeruginosa [IMP] IHMA 576602	IMP | *Pseudomonas aeruginosa* | *Pseudomonas* spp.	8.97×10^{8}<br>7.30×10^{8}<br>7.70×10^{8}	7.99×10^{8}	9/9 (100%)	1.33×10^{9}<br>1.14×10^{9}<br>1.30×10^{9}	1.26×10^{9}	9/9 (100%)
Stenotrophomonas maltophilia ATCC 13636	*Stenotrophomonas maltophilia*	3.83×10^{8}<br>5.23×10^{8}<br>4.37×10^{8}	4.48×10^{8}	9/9 (100%)	1.23×10^{9}<br>9.47×10^{8}<br>8.27×10^{8}	1.00×10^{9}	9/9 (100%)
Proteus mirabilis ATCC 12453	*Enterobacteriaceae / Morganellaceae* | *Proteus* spp.	2.14×10^{9}<br>2.35×10^{9}<br>2.30×10^{9}	2.26×10^{9}	9/9 (100%)	2.28×10^{9}<br>1.80×10^{9}<br>2.28×10^{9}	2.12×10^{9}	9/9 (100%)
Haemophilus influenzae ATCC 9007	*Haemophilus influenzae*	2.12×10^{9}<br>2.32×10^{9}<br>1.96×10^{9}	2.13×10^{9}	9/9 (100%)	1.37×10^{9}<br>2.42×10^{9}<br>1.32×10^{9}	1.70×10^{9}	9/9 (100%)
Neisseria meningitidis ATCC 43744	*Neisseria meningitidis*	2.39×10^{8}<br>2.36×10^{8}<br>8.93×10^{7}	1.88×10^{8}	9/9 (100%)	1.43×10^{8}<br>4.10×10^{8}<br>3.07×10^{8}	2.87×10^{8}	9/9 (100%)

[a]One OXA false positive was observed during initial testing of *Serratia marcescens*. An additional set of replicates was tested resulting in 12 total replicates.

Abbreviation: CFU, colony-forming units.

Supplemental Table S5. Mean concentration of blood culture organisms used for testing different media bottles

Gram-Negative (On-Panel) Organism[a]	Expected Result	Strain ID	Average Positive Blood Culture Concentration (CFU/mL)
Acinetobacter baumannii	*Acinetobacter* spp., *Acinetobacter baumannii*, OXA	IHMA 128307	3.03×10^{8}
Acinetobacter lwoffii	*Acinetobacter* spp.	ATCC 15309	2.05×10^{8}
Citrobacter freundii	*Enterobacteriaceae/Morganellaceae*, *Citrobacter* spp., VIM	IHMA 549813	1.03×10^{9}
Citrobacter amalonaticus	*Enterobacteriaceae/Morganellaceae*, *Citrobacter* spp.	ATCC 25405	9.87×10^{9}
Enterobacter cloacae	*Enterobacteriaceae/Morganellaceae*, *Enterobacter* spp.	ATCC 35030	1.16×10^{9}
Enterobacter aerogenes	*Enterobacteriaceae/Morganellaceae*, *Enterobacter* spp.	ATCC 35029	9.50×10^{9}
Proteus mirabilis	*Enterobacteriaceae/Morganellaceae*, *Proteus* spp.	ATCC 12453	6.64×10^{8}
Proteus vulgaris	*Enterobacteriaceae/Morganellaceae*, *Proteus* spp.	ATCC 29905	4.54×10^{8}
Salmonella bongori	*Enterobacteriaceae/Morganellaceae*, *Salmonella* spp.	ATCC 43975	1.42×10^{9}
Salmonella enterica subsp. *arizonae*	*Enterobacteriaceae/Morganellaceae*, *Salmonella* spp.	ATCC 13314	1.67×10^{9}
Pseudomonas aeruginosa	*Pseudomonas* spp., *Pseudomonas aeruginosa*, IMP	IHMA 576602	4.88×10^{8}
Pseudomonas mendocina	*Pseudomonas* spp.	ATCC 25411	5.24×10^{8}
Klebsiella oxytoca	*Enterobacteriaceae/Morganellaceae*, *Klebsiella oxytoca*, CTX-M	IHMA 683079	7.29×10^{8}
Klebsiella pneumoniae	*Enterobacteriaceae/Morganellaceae*, *Klebsiella pneumoniae*, KPC	IHMA 629630	6.61×10^{8}
Klebsiella variicola	*Enterobacteriaceae/Morganellaceae*, *Klebsiella variicola*	Clinical Isolate V0512	1.28×10^{9}
Escherichia coli	*Enterobacteriaceae/Morganellaceae*, *Escherichia coli*, MCR	NCTC 13846	1.17×10^{9}
Serratia marcescens	*Enterobacteriaceae/Morganellaceae*, *Serratia marcescens*, SME	IHMA 1642209	1.34×10^{9}
Morganella morganii	*Enterobacteriaceae/Morganellaceae*, *Morganella morganii*, NDM	IHMA 605873	1.28×10^{9}
Haemophilus influenzae	*Haemophilus influenzae*	ATCC 9007	6.70×10^{8}

Neisseria meningitidis	*Neisseria meningitidis*	ATCC 43744	3.07×10^{8}
Stenotrophomonas maltophilia	*Stenotrophomonas maltophilia*	ATCC 700269	4.18×10^{8}

Gram-Positive (Off-panel) Organism[a]	Expected Result	Strain ID	Average Positive Blood Culture Concentration (CFU/mL)
Enterococcus faecalis	No Target Detected	ATCC 51575	7.51×10^{8}
Enterococcus faecium	No Target Detected	ATCC 700221	4.12×10^{8}
Staphylococcus aureus (MRSA)	No Target Detected	ATCC BAA-2312	2.07×10^{8}
Staphylococcus epidermidis (MRSE)	No Target Detected	ATCC 35984	2.26×10^{8}
Streptococcus agalactiae	No Target Detected	ATCC 12386	7.50×10^{8}
Streptococcus constellatus	No Target Detected	ATCC 27823	5.25×10^{8}
Bacillus subtilis	No Target Detected	ATCC 19659	8.50×10^{7}
Bacillus cereus[b]	No Target Detected	ATCC 10702	4.40×10^{8}
Corynebacterium diphtheriae	No Target Detected	ATCC 27010	3.74×10^{8}
Corynebacterium striatum	No Target Detected	ATCC 43735	3.48×10^{8}
Listeria monocytogenes	No Target Detected	ATCC 15313	7.58×10^{8}

[a] Genus and species taxonomies are ever evolving based on the latest research. The strain ID is a unique identifier from the supplier and should be the reference used for that material.

[b] Concentration of *Bacillus cereus* representing single blood bottle culture in VersaTREK™ REDOX™2 EZ Draw™ Media.

Abbreviation: CFU, colony-forming units.

Supplemental Table S6. LIAISON PLEX® Gram-Negative Blood Culture Assay media equivalency

Manufacturer System	Blood Culture Bottle Manufacturer	Blood Culture Bottle Type[a]	Number of Inoculated Bottles		
			Gram-Negative Bacteria (Positive Detection)	Gram-Positive Bacteria (Positive Detection)	Negative Blood Matrix[b] (Positive Detection)
**bioMérieux BACT/ALERT® 3D System**	**bioMérieux BACT/ALERT®**	BACT/ALERT® SA (Aerobic)	21/21	0/10	0/2
		BACT/ALERT® SN (Anaerobic)	58/58	0/10	0/2
		BACT/ALERT® FN Plus (Anaerobic)	21/21	0/10	0/2
		BACT/ALERT® PF Plus (Pediatric)	63/63	0/10	0/2
**N/A[c]**	**Becton Dickinson[a] BACTEC™**	BACTEC™ Standard	21/21	0/10	0/2
		BACTEC™ Plus (Aerobic)	21/21	0/10	0/2
		BACTEC™ Standard (Anaerobic)	50/50	0/10	0/2
		BACTEC™ Plus (Anaerobic)	58/58	0/10	0/2
		BACTEC™ Peds Plus (Pediatric)	63/63	0/10	0/2
		BACTEC™ Lytic (Anaerobic)	20/20	0/10	0/2
**N/A[c]**	**Thermo Scientific[a] VersaTREK™**	REDOX™ 1 EZ Draw™ (Aerobic)	60/60	0/10	0/2
		REDOX™ 2 EZ Draw™ (Anaerobic)	50/50	0/10	0/2
**Number of Expected Accurate Calls/Total**			**506/506 (100%)**	**120/120 (100%)**	**24/24 (100%)**
**Number of Independent Blood Bottles**			**Overall Accuracy=650/650 (100%)**		

[a]bioMérieux BACT/ALERT® FA Plus was established as the standard media for the clinical evaluation and for all other analytical studies, *e.g.*, growth and detection and analytical reactivity/ inclusivity verification testing, and was not included in the matrix equivalency study.

[b]Negative Blood Matrix (NBM) was tested using two independent blood inoculations per bottle type and each NBM sample was tested in replicates of three (total six replicates per bottle type) per study design; 72 total runs from 24 total bottle inoculations.

[c]For gram-negative blood culture preparation, BACTEC™ and VersaTREK™ systems were not available and corresponding media bottles were placed in a standard laboratory incubator with a shaker for growth. For gram-positive blood culture growth, only VersaTREK™ bottles needed to be placed in a standard laboratory incubator with a shaker in lieu of VersaTREK™ system; BACT/ALERT® and BACTEC™ blood bottles were grown using corresponding automated blood culture systems up to bottle ring positivity.

Supplemental Table S7. LIAISON PLEX® BCN Assay Cross-Reactive Organisms Summary

Organism	Positivity[a]	Inclusive	Cross-reacting
**Not Reported to Cause Bloodstream Infections**			
Lelliottia amnigena	100%	*Enterobacteriaceae / Morganellaceae*	*Enterobacter* spp.
Pseudomonas paraeruginosa	100%	*Pseudomonas* spp.	*Pseudomonas aeruginosa*
Serratia nevei	100%	*Enterobacteriaceae / Morganellaceae*	*Serratia marcescens*
Serratia entomophila	100%	*Enterobacteriaceae / Morganellaceae*	*Serratia marcescens*
Serratia ureilytica	100%	*Enterobacteriaceae / Morganellaceae*	*Serratia marcescens*
Klebsiella quasivariicola	100%	*Enterobacteriaceae / Morganellaceae*	*Klebsiella pneumoniae*
**Rare/Not Primarily Associated with Bloodstream Infections**			
**Organism**	**Positivity[a]**	**Inclusive**	**Cross-reacting**
Haemophilus aegyptius	100%	None	*Haemophilus influenzae*
Klebsiella michiganensis	100%	*Enterobacteriaceae / Morganellaceae*	*Klebsiella oxytoca*
**Rare/Opportunistic with Some Documented Bloodstream Infection Cases**			
**Organism**	**Positivity[a]**	**Inclusive**	**Cross-reacting**
Klebsiella grimontii	100%	*Enterobacteriaceae / Morganellaceae*	*Klebsiella oxytoca*
Klebsiella quasipneumoniae	100%	*Enterobacteriaceae / Morganellaceae*	*Klebsiella pneumoniae*
Haemophilus parahaemolyticus	33%	None	*Enterobacteriaceae / Morganellaceae*

[a]Each off-panel organism was tested in triplicate assay cartridges.

Supplemental Table S8. LIAISON PLEX BCN Assay on-panel competitive inhibition summary

On-Panel High Concentration Target	Positivity	On-Panel Low Concentration Target	Concentration (CFU/mL)	Positivity
Acinetobacter baumannii $2.09×10^8$ CFU/mL	100%	*Citrobacter freundii*	$9.63×10^8$	100%
	100%	*Enterobacter cloacae*	$1.46×10^9$	100%
	100%	*Escherichia coli*	$1.70×10^9$	100%
	100%	*Haemophilus influenzae*	$2.12×10^9$	100%
	100%	*Klebsiella oxytoca*	$4.53×10^8$	100%
	100%	*Klebsiella pneumoniae*	$1.33×10^9$	100%
	100%	*Neisseria meningitidis*	$2.39×10^8$	100%
	100%	*Proteus mirabilis*	$2.14×10^9$	100%
	100%	*Pseudomonas aeruginosa*	$8.97×10^8$	100%
Citrobacter freundii $1.76×10^9$ CFU/mL	100%	*Acinetobacter baumannii*	$2.13×10^8$	100%
	100%	*Enterobacter cloacae*	$1.46×10^9$	100%
	100%	*Escherichia coli*	$1.70×10^9$	100%
	100%	*Haemophilus influenzae*	$2.12×10^9$	100%
	100%	*Klebsiella oxytoca*	$4.53×10^8$	100%
	100%	*Klebsiella pneumoniae*	$1.33×10^9$	100%
	100%	*Neisseria meningitidis*	$2.39×10^8$	100%
	100%	*Proteus mirabilis*	$2.14×10^9$	100%
	100%	*Pseudomonas aeruginosa*	$8.97×10^8$	100%
Enterobacter cloacae $1.68×10^9$ CFU/mL	100%	*Acinetobacter baumannii*	$2.13×10^8$	100%
	100%	*Citrobacter freundii*	$9.63×10^8$	100%
	100%	*Escherichia coli*	$1.70×10^9$	100%
	100%	*Haemophilus influenzae*	$2.12×10^9$	100%
	100%	*Klebsiella oxytoca*	$4.53×10^8$	100%
	100%	*Klebsiella pneumoniae*	$1.33×10^9$	100%
	100%	*Neisseria meningitidis*	$2.39×10^8$	100%
	100%	*Proteus mirabilis*	$2.14×10^9$	100%
	100%	*Pseudomonas aeruginosa*	$8.97×10^8$	100%
Escherichia coli $1.45×10^9$ CFU/mL	100%	*Acinetobacter baumannii*	$2.13×10^8$	100%
	100%	*Citrobacter freundii*	$9.63×10^8$	100%
	100%	*Enterobacter cloacae*	$1.46×10^9$	100%
	100%	*Haemophilus influenzae*	$2.12×10^9$	100%
	100%	*Klebsiella oxytoca*	$4.53×10^8$	100%
	100%	*Klebsiella pneumoniae*	$1.33×10^9$	100%
	100%	*Neisseria meningitidis*	$2.39×10^8$	100%
	100%	*Proteus mirabilis*	$2.14×10^9$	100%
	100%	*Pseudomonas aeruginosa*	$8.97×10^8$	100%
Haemophilus influenzae $1.37×10^9$ CFU/mL	100%	*Acinetobacter baumannii*	$2.13×10^8$	100%
	100%	*Citrobacter freundii*	$9.63×10^8$	100%
	100%	*Enterobacter cloacae*	$1.46×10^9$	100%
	100%	*Escherichia coli*	$1.70×10^9$	100%
	100%	*Klebsiella oxytoca*	$4.53×10^8$	100%

	100%	*Klebsiella pneumoniae*	1.33×10^9	100%
	100%	*Neisseria meningitidis*	2.39×10^8	100%
	100%	*Proteus mirabilis*	2.14×10^9	100%
	100%	*Pseudomonas aeruginosa*	8.97×10^8	100%
Klebsiella oxytoca 5.30×10^8 CFU/mL	100%	*Acinetobacter baumannii*	2.13×10^8	100%
	100%	*Citrobacter freundii*	9.63×10^8	100%
	100%	*Enterobacter cloacae*	1.46×10^9	100%
	100%	*Escherichia coli*	1.70×10^9	100%
	100%	*Haemophilus influenzae*	2.12×10^9	100%
	100%	*Klebsiella pneumoniae*	1.33×10^9	100%
	100%	*Neisseria meningitidis*	2.39×10^8	100%
	100%	*Proteus mirabilis*	2.14×10^9	100%
	100%	*Pseudomonas aeruginosa*	8.97×10^8	100%
Klebsiella pneumoniae 1.55×10^9 CFU/mL	100%	*Acinetobacter baumannii*	2.13×10^8	100%
	100%	*Citrobacter freundii*	9.63×10^8	100%
	100%	*Enterobacter cloacae*	1.46×10^9	100%
	100%	*Escherichia coli*	1.70×10^9	100%
	100%	*Haemophilus influenzae*	2.12×10^9	100%
	100%	*Klebsiella oxytoca*	4.53×10^8	100%
	100%	*Neisseria meningitidis*	2.39×10^8	100%
	100%	*Proteus mirabilis*	2.14×10^9	100%
	100%	*Pseudomonas aeruginosa*	8.97×10^8	100%
Neisseria meningitidis 1.43×10^8 CFU/mL	100%	*Acinetobacter baumannii*	2.13×10^8	100%
	100%	*Citrobacter freundii*	9.63×10^8	100%
	100%	*Enterobacter cloacae*	1.46×10^9	100%
	100%	*Escherichia coli*	1.70×10^9	100%
	100%	*Haemophilus influenzae*	2.12×10^9	100%
	100%	*Klebsiella oxytoca*	4.53×10^8	100%
	100%	*Klebsiella pneumoniae*	1.33×10^9	100%
	100%	*Proteus mirabilis*	2.14×10^9	100%
	100%	*Pseudomonas aeruginosa*	8.97×10^8	100%
Proteus mirabilis 2.28×10^9 CFU/mL	100%	*Acinetobacter baumannii*	2.13×10^8	100%
	100%	*Citrobacter freundii*	9.63×10^8	100%
	100%	*Enterobacter cloacae*	1.46×10^9	100%
	100%	*Escherichia coli*	1.70×10^9	100%
	100%	*Haemophilus influenzae*	2.12×10^9	100%
	100%	*Klebsiella oxytoca*	4.53×10^8	100%
	100%	*Klebsiella pneumoniae*	1.33×10^9	100%
	100%	*Neisseria meningitidis*	2.39×10^8	100%
	100%	*Pseudomonas aeruginosa*	8.97×10^8	100%
Pseudomonas aeruginosa 1.33×10^9 CFU/mL	100%	*Acinetobacter baumannii*	2.13×10^8	100%
	100%	*Citrobacter freundii*	9.63×10^8	100%
	100%	*Enterobacter cloacae*	1.46×10^9	100%
	100%	*Escherichia coli*	1.70×10^9	100%

	100%	*Haemophilus influenzae*	2.12×10^9	100%
	100%	*Klebsiella oxytoca*	4.53×10^8	100%
	100%	*Klebsiella pneumoniae*	1.33×10^9	100%
	100%	*Neisseria meningitidis*	2.39×10^8	100%
	100%	*Proteus mirabilis*	2.14×10^9	100%

Abbreviation: CFU, colony-forming units.

Supplemental Table S9. LIAISON PLEX® Gram-Negative Blood Culture Assay microbial interference summary

On-Panel Low Concentration Target	Positivity	Off-Panel High Concentration Target	Off-Panel Target Conc. (CFU/mL)	Positivity
Acinetobacter baumannii 2.13×10^8 CFU/mL	100%	*Bacillus cereus*	3.43×10^8	0%
	100%	*Clostridium perfringens*	1.06×10^8	0%
	100%	*Corynebacterium striatum*	1.47×10^9	0%
	100%	*Cutibacterium (Propionibacterium) acnes*	5.70×10^8	0%
	100%	*Staphylococcus aureus*	2.16×10^9	0%
	100%	*Staphylococcus epidermidis*	5.70×10^8	0%
	100%	*Streptococcus mitis*	6.00×10^8	0%
Citrobacter freundii 9.63×10^8 CFU/mL	100%	*Bacillus cereus*	3.43×10^8	0%
	100%	*Clostridium perfringens*	1.06×10^8	0%
	100%	*Corynebacterium striatum*	1.47×10^9	0%
	100%	*Cutibacterium (Propionibacterium) acnes*	5.70×10^8	0%
	100%	*Staphylococcus aureus*	2.16×10^9	0%
	100%	*Staphylococcus epidermidis*	5.70×10^8	0%
	100%	*Streptococcus mitis*	6.00×10^8	0%
Enterobacter cloacae 1.46×10^9 CFU/mL	100%	*Bacillus cereus*	3.43×10^8	0%
	100%	*Clostridium perfringens*	1.06×10^8	0%
	100%	*Corynebacterium striatum*	1.47×10^9	0%
	100%	*Cutibacterium (Propionibacterium) acnes*	5.70×10^8	0%
	100%	*Staphylococcus aureus*	2.16×10^9	0%
	100%	*Staphylococcus epidermidis*	5.70×10^8	0%
	100%	*Streptococcus mitis*	6.00×10^8	0%
Escherichia coli 1.70×10^9 CFU/mL	100%	*Bacillus cereus*	3.43×10^8	0%
	100%	*Clostridium perfringens*	1.06×10^8	0%
	100%	*Corynebacterium striatum*	1.47×10^9	0%
	100%	*Cutibacterium (Propionibacterium) acnes*	5.70×10^8	0%
	100%	*Staphylococcus aureus*	2.16×10^9	0%
	100%	*Staphylococcus epidermidis*	5.70×10^8	0%
	100%	*Streptococcus mitis*	6.00×10^8	0%
Haemophilus influenzae 2.12×10^9 CFU/mL	100%	*Bacillus cereus*	3.43×10^8	0%
	100%	*Clostridium perfringens*	1.06×10^8	0%
	100%	*Corynebacterium striatum*	1.47×10^9	0%
	100%	*Cutibacterium (Propionibacterium) acnes*	5.70×10^8	0%
	100%	*Staphylococcus aureus*	2.16×10^9	0%
	100%	*Staphylococcus epidermidis*	5.70×10^8	0%
	100%	*Streptococcus mitis*	6.00×10^8	0%
Klebsiella oxytoca 4.53×10^8 CFU/mL	100%	*Bacillus cereus*	3.43×10^8	0%
	100%	*Clostridium perfringens*	1.06×10^8	0%

	100%	*Corynebacterium striatum*	1.47×10^9	0%
	100%	*Cutibacterium (Propionibacterium) acnes*	5.70×10^8	0%
	100%	*Staphylococcus aureus*	2.16×10^9	0%
	100%	*Staphylococcus epidermidis*	5.70×10^8	0%
	100%	*Streptococcus mitis*	6.00×10^8	0%
Klebsiella pneumoniae 1.33×10^9 CFU/mL	100%	*Bacillus cereus*	3.43×10^8	0%
	100%	*Clostridium perfringens*	1.06×10^8	0%
	100%	*Corynebacterium striatum*	1.47×10^9	0%
	100%	*Cutibacterium (Propionibacterium) acnes*	5.70×10^8	0%
	100%	*Staphylococcus aureus*	2.16×10^9	0%
	100%	*Staphylococcus epidermidis*	5.70×10^8	0%
	100%	*Streptococcus mitis*	6.00×10^8	0%
Neisseria meningitidis 2.39×10^8 CFU/mL	100%	*Bacillus cereus*	3.43×10^8	0%
	100%	*Clostridium perfringens*	1.06×10^8	0%
	100%	*Corynebacterium striatum*	1.47×10^9	0%
	100%	*Cutibacterium (Propionibacterium) acnes*	5.70×10^8	0%
	100%	*Staphylococcus aureus*	2.16×10^9	0%
	100%	*Staphylococcus epidermidis*	5.70×10^8	0%
	100%	*Streptococcus mitis*	6.00×10^8	0%
Proteus mirabilis 2.14×10^9 CFU/mL	100%	*Bacillus cereus*	3.43×10^8	0%
	100%	*Clostridium perfringens*	1.06×10^8	0%
	100%	*Corynebacterium striatum*	1.47×10^9	0%
	100%	*Cutibacterium (Propionibacterium) acnes*	5.70×10^8	0%
	100%	*Staphylococcus aureus*	2.16×10^9	0%
	100%	*Staphylococcus epidermidis*	5.70×10^8	0%
	100%	*Streptococcus mitis*	6.00×10^8	0%
Pseudomonas aeruginosa 8.97×10^8 CFU/mL	100%	*Bacillus cereus*	3.43×10^8	0%
	100%	*Clostridium perfringens*	1.06×10^8	0%
	100%	*Corynebacterium striatum*	1.47×10^9	0%
	100%	*Cutibacterium (Propionibacterium) acnes*	5.70×10^8	0%
	100%	*Staphylococcus aureus*	2.16×10^9	0%
	100%	*Staphylococcus epidermidis*	5.70×10^8	0%
	100%	*Streptococcus mitis*	6.00×10^8	0%

Abbreviation: CFU, colony-forming units.
